# Dynamic evolution of transient receptor potential vanilloid (TRPV) ion channel family with numerous gene duplications and losses

**DOI:** 10.3389/fendo.2022.1013868

**Published:** 2022-11-01

**Authors:** Marina Morini, Christina A. Bergqvist, Juan F. Asturiano, Dan Larhammar, Sylvie Dufour

**Affiliations:** ^1^ Laboratory Biology of Aquatic Organisms and Ecosystems (BOREA), National Museum of Natural History (MNHN), CNRS, IRD, Sorbonne University, Paris, France; ^2^ Grupo de Acuicultura y Biodiversidad, Instituto de Ciencia y Tecnología Animal, Universitat Politècnica de València, Valencia, Spain; ^3^ Department of Medical Cell Biology, Science for Life Laboratory, Uppsala University, Uppsala, Sweden

**Keywords:** transient receptor potential channel, TRPV, metazoans, vertebrates, evolution, phylogeny, synteny, tetraploidization

## Abstract

The transient receptor potential vanilloid (TRPV) ion channel family is involved in multiple sensory and physiological functions including thermosensing and temperature-dependent neuroendocrine regulation. The objective of the present study was to investigate the number, origin and evolution of TRPV genes in metazoans, with special focus on the impact of the vertebrate whole-genome duplications (WGD). Gene searches followed by phylogenetic and synteny analyses revealed multiple previously undescribed TRPV genes. The common ancestor of Cnidaria and Bilateria had three TRPV genes that became four in the deuterostome ancestor. Two of these were lost in the vertebrate ancestor. The remaining two genes gave rise to two TRPV subfamilies in vertebrates, consisting of subtypes 1, 2, 3, 4, 9 and 5, 6, 7, 8, respectively. This gene expansion resulted from the two basal vertebrate WGD events (1R and 2R) and three local duplications before the radiation of gnathostomes. TRPV1, 4 and 5 have been retained in all gnathostomes investigated, presumably reflecting important functions. TRPV7 and 8 have been lost independently in various lineages but are still retained in cyclostomes, actinistians (coelacanth), amphibians, prototherians and basal actinopterygians (Polypteridae). TRPV3 and 9 are present in extant elasmobranchs, while TRPV9 was lost in the osteichthyan ancestor and TRPV3 in the actinopterygian ancestor. The coelacanth has retained the ancestral osteichthyan repertoire of TRPV1, 3, 4, 5, 7 and 8. TRPV2 arose in the tetrapod ancestor. Duplications of TRPV5 occurred independently in various lineages, such as cyclostomes, chondrichthyans, anuran amphibians, sauropsids, mammals (where the duplicate is called TRPV6), and actinopterygians (Polypteridae and Esocidae). After the teleost-specific WGD (3R) only TRPV1 retained its duplicate, whereas TRPV4 and 5 remained as single genes. Both 3R-paralogs of TRPV1 were kept in some teleost species, while one paralog was lost in others. The salmonid-specific WGD (4R) duplicated TRPV1, 4, and 5 leading to six TRPV genes. The largest number was found in *Xenopus tropicalis* with no less than 15 TRPV genes. This study provides a comprehensive evolutionary scenario for the vertebrate TRPV family, revealing additional TRPV types and proposing a phylogeny-based classification of TRPV across metazoans.

## 1 Introduction

The discovery of receptors for temperature was awarded the Nobel Prize in Physiology or Medicine to David Julius and Ardem Patapoutian in 2021, highlighting the importance of these receptors for the interactions of organisms with their environment. These receptors belong to the transient receptor potential (TRP) channel superfamily which is a group of multifunctional cell membrane proteins involved in many sensory and physiological functions and expressed in various tissues (1–3). The TRP superfamily members have been classified into nine TRP families in metazoans, in alphabetical order named TRPA (TRP ankyrin), TRPC (TRP canonical), TRPM (TRP melastatin), TRPML (TRP mucolipin), TRPN (TRP nompC, or no mechanoreceptor potential C), TRPP (TRP polycystin or polycystic kidney disease), TRPS (TRP soromelastatin), TRPV (TRP vanilloid), and TRPVL (TRP vanilloid-like) (4). Notably, ankyrin domains are found not only in the TRPA family but also in the TRPC and TRPV families.

The founding member of the TRPV family, TRPV1, was first identified in rat neurons, based on its sensitivity to both heat and capsaicin (5, 6). Subsequently five additional members were identified in mammals and named TRPV2 to TRPV6 (1, 7). The TRPV members have been implicated in sensory transduction in both vertebrates and non-vertebrates. In addition to temperature and pain, they are involved in osmotic regulation and maintenance of calcium homeostasis. They have also been linked to inflammatory, immunoendocrine processes and viral infection (2, 8–13).

Among the six mammalian family members, TRPV1 to 4 are classified as thermosensitive (thermoTRPV channels) and are well conserved among each other with 40–50% sequence identity between subtypes (14). TRPV4 has broad expression and has been implicated in a variety of functions including osmotic regulation and chemical and mechanic stimuli (15). Mammalian TRPV5 and TRPV6 display 75% identity to each other but only ∼30% to TRPV1 to 4 (16). TRPV5 and TRPV6 are not considered to be thermoreceptors and exhibit selectivity for Ca^2+^ over other cations (17). TRPV5 has prominent roles in kidneys (18) and is also co-expressed with vasopressin and oxytocin in rat hypothalamic neurons (19). TRPV6 has been reported to be predominantly involved in Ca^2+^ absorption in the small intestine (18).

Among non-mammalian vertebrates, TRPV family members have been identified in birds as well as ectothermic vertebrates such as amphibians, sauropsids and fishes. However, their physiological roles remain largely unexplored and only a few studies have investigated TRPV in fish. Three TRPVs have been described so far in teleosts, TRPV1, TRPV4 and TRPV6 (20). In rainbow trout, TRPV1 and TRPV4 are expressed in a wide range of tissues, including in the pineal; both TRPV1 and TRPV4 are involved in the regulation of melatonin secretion *in vitro*, and at least TRPV1 is involved in thermosensing (21). In Atlantic salmon (*Salmo salar*), TRPV1 and TRPV4 are tightly linked to thermal sensing during behavioral fever (22). In zebrafish, TRPV1 and TRPV4 are expressed in various sensory organs (23, 24).

In the same year as TRPV1 was described in rat, the TRPV homolog named OSM-9 (OSM for osmotic) was characterized in an ecdysozoan protostome, *Caenorhabditis elegans* (25). Subsequently, four additional OSM-9-like members were identified and named OCR 1–4 (OCR for Osm-9 and Capsaicin receptor-Related). Like OSM-9, these display the typical TRPV structure with multiple ankyrin repeat domains (26). In another ecdysozoan, the fruit fly *Drosophila melanogaster*, two members of the TRPV family have been identified: Nan (Nanchung) and IAV (inactive, also called putative CG4536 protein) (16, 27). The *C. elegans* TRPV channels OSM-9 and OCR-2 are essential for some forms of mechano-sensation (osmosensation, nose touch), whereas the *Drosophila* TRPV channels NAN and IAV are essential for sound transduction by the antennal chordotonal organ (28). In *C. elegans* TRPV channels are expressed in neuroendocrine cells and promote neurotransmitter release (29). TRPV homologs have been found in cnidarians, demonstrating that the TRPV family originated before the emergence of bilaterians (30).

In the current context of climate change and global warming, considering that TRPV channels may be involved in temperature sensing and subsequent regulation of physiological functions, there is a rising interest in investigation of TRPV channels in various species. The objective of the present study was to investigate the evolution of the TRPV family in metazoans, with special focus on the impact of genome and gene duplications as well as of gene losses in vertebrates. In the ancestor of gnathostomes (jawed vertebrates), two whole-genome duplications (WGD) took place (31, 32), referred to as “1R” and “2R” for the first and second rounds of WGD. These events increased the number of genes and thereby provided the vertebrates with new potential molecular tools (33). A third WGD occurred later in the teleost lineage, referred to as “3R” or the teleost-specific whole genome duplication (TWGD) (34). Further WGDs happened more recently in some teleost lineages such as salmonids (35), referred to as “4R”, or “SWGD” (salmonid-specific whole genome duplication).

After a WGD, some of the duplicates may be lost while others remain and may evolve new functions (neofunctionalization). In other instances, the two duplicates may partition the functions of the ancestral single-copy gene (subfunctionalization). These WGD events as well as potential independent gene duplications, as reported for instance for voltage-gated sodium channels (36), likely impacted also the number of TRPV genes during vertebrate evolution. We therefore performed TRPV gene searches and phylogenetic and synteny analyses in vertebrates as well as other metazoans representing key phylogenetic positions. This enabled us to reveal three additional TRPV types of ancient origin in vertebrates and to identify several previously undescribed TRPV paralogs in various lineages. By combining sequence-based phylogenetic analyses with investigation of conserved synteny and paralogons (i.e., groups of related chromosomal regions) resulting from WGD events, we have been able to deduce a likely scenario for the evolution of the TRPV family in metazoans, with special focus on vertebrates. We propose here a phylogeny-based classification of the repertoire of TRPV genes across metazoans including vertebrates as a framework for future investigations of thermoreceptors in various metazoan biological models (from corals to vertebrates) of relevance for biodiversity conservation, agronomy/aquaculture, socio-economics, and even biomedical studies.

## 2 Material and methods

### 2.1 Gene search for TRPV sequences

TRPV sequences from representative species of various metazoan groups were retrieved using BLAST search against NCBI (https://www.ncbi.nlm.nih.gov/) or Ensembl (Ensembl release 103, https://www.ensembl.org/index.html) genome databases. The TBLASTN algorithm of the Ensembl Genome Browser website was also used to identify non-annotated TRPV genes in the genomes of the species included in this study, to search for pseudogenes or to confirm gene loss. TBLASTN algorithm of the CLC Main Workbench 21.0.3 software (CLC bio, Aarhus, Denmark) was used to manually complete some sequences of TRPV genes, or to confirm gene loss. BLAST searches in NCBI were done to identify non-annotated TRPV genes retrieved from the Ensembl database. When a TRPV ortholog was missing in any of the species included, potential pseudogenes were searched. After identification of TRPV pseudogene, the sequence was extended by manual inspection. Sequence references are provided in **Supplementary Table S1**.

### 2.2 Phylogenetic analysis of TRPV family in metazoans

Phylogenetic analyses were performed on representative species at key phylogenetic positions among metazoans: cnidarians (*Orbicella faveolata*; *Acropora millepora*); protostomes (two lophotrochozoans: a mollusk, the scallop *Pecten maximus*; an annelid, the vent worm *Lamellibrachia satsuma;* two ecdysozoans: a nematode *Caenorhabditis elegans* and the fruit fly *Drosophila melanogaster*); non-chordate deuterostomes (a hemichordate *Saccoglossus kowalevskii*; three echinoderms: the starfish *Asterias rubens*, the sea urchin *Strongylocentrotus purpurea* and the sea cucumber *Apostichopus japonicus)*; non-vertebrate chordates (a cephalochordate *Branchiostoma floridae*; three urochordates: the ascidians *Ciona intestinalis* and *Ciona savignyi* and the sea pineapple *Halocynthia roretzi)*; a cyclostome, the sea lamprey (*Petromyzon marinus*); chondrichthyans (holocephalan: the elephant shark *Callorhinchus milii*; three elasmobranchs: the spotted catshark *Scyliorhinus canicula*, the whale shark *Rhincodon typus* and the thorny skate *Amblyraja radiata*); a representative for an early diverging sarcopterygian lineage, the actinistian coelacanth (*Latimeria chalumnae*); representatives of tetrapods including mammals (proto-, meta- and eu-therians), sauropsids (squamates, chelonians, crocodilians and birds) and amphibians; non-teleost actinopterygians (two Polypteridae, the reedfish *Erpetoichthys calabaricus*, and the bichir *Polypterus senegalus;* an holostean, the spotted gar *Lepisosteus oculatus*); two representatives for basally diverging teleost lineages (an elopomorph, the European eel *Anguilla anguilla* and an osteoglossomorph, the Asian bonytongue *Scleropages formosus*), and various other teleosts.

Rooted phylogenetic trees were constructed with amino acid sequences of TRPV (for accession/ID number, see **Supplementary Table S1**). For the global TRPV phylogeny tree, some TRPA, TRPM, TRPN and TRPP sequences from vertebrate and non-vertebrate species were included and the tree was rooted by mouse (*Mus musculus*) and fruit fly TRPP sequences. The sequences were first aligned using Clustal Omega (37) with SeaView 5.0.1 software (http://doua.prabi.fr/software/seaview) or MEGA X (38), and then manually adjusted. The JTT (Jones, Taylor and Thornton) protein substitution matrix of the resulting alignment was determined using ProTest software (39). The phylogenetic trees of TRPV were constructed based on the sequence alignments, using the RAXML program (Randomized Axelerated Maximum Likelihood; 40) with 1000 bootstrap replicates, and subsequently visualized using Figtree 1.4.4 (http://tree.bio.ed.ac.uk/).

### 2.3 Synteny analyses of the TRPV family in vertebrates

Synteny analyses of TRPV genomic regions were performed for vertebrate species at key phylogenetic positions and included a cyclostome (sea lamprey), chondrichthyans (elephant shark and spotted catshark), sarcopterygians (coelacanth and tetrapods) and actinopterygians (reedfish and/or spotted gar and several teleosts). The following strategies were used: neighboring genes were first identified using Genomicus PhyloView of Genomicus v100.01, using human or platypus (*Ornithorhynchus anatinus*) as starting point/template/reference. BLAST analyses in ENSEMBL and NCBI were performed to search for potential paralogs of the neighboring genes, or to identify non-annotated neighboring genes in the above-mentioned genomes.

Due to the absence of the European eel genome in Ensembl and Genomicus databases, neighboring genomic regions of the TRPVs were first characterized manually in the European eel genome (GCF_013347855.1, fAngAng1 genome, Future Genomics Technologies B.V., Leiden, Netherland), using CLC Main Workbench 21.0.3 software (CLC bio, Aarhus, Denmark). A previous European eel draft genome from nanopore sequencing reads (41) was also used. The locations and exon-intron organization of genes in the same scaffolds as the TRPV genes were first predicted using the GENSCAN Web Server (http://genes.mit.edu/GENSCAN.html). BLAST analyses of the predicted genes were performed in the NCBI database to identify the neighbors of the TRPV genes. As TRPV genes were located in short scaffolds in the coelacanth draft genome (GCA_000225785.1, LatCha1), the TBLASTN algorithm of the CLC Main Workbench 21.0.3 software was used to identify the neighboring genes. As Western clawed frog (*Xenopus tropicalis*) TRPV5 and TRPV5-neighbouring genes were located on small scaffolds in the previous genome assembly available in the Ensembl database (Xenopus_tropicalis_v9.1, GCF_000004195.3), a more recent version in the NCBI database was used (UCB_Xtro_10.0, GCF_000004195.4). TRPV neighboring gene references and locations are provided in **Supplementary Table S2**.

### 2.4 Paralogon analyses of the TRPV family in vertebrates

For paralogon (synteny block) analyses, each gnathostome TRPV genomic region was used separately as starting point (i.e., TRPV1/TRPV2/TRPV3/TRPV9, TRPV4, TRPV5/TRPV6, and TRPV7/TRPV8 genomic region, respectively). The spotted gar was used as starting point for TRPV1, 2, 3, 4, 9, gene lists of the genomic regions 10 Mb upstream and downstream of the TRPV gene family members were downloaded using the Biomart function in Ensembl version 80. From the gene lists, gene families with at least two members were selected. Additional members were searched with BLAST and those having three or four members were included (see section 3.3.5). For TRPV5/6, 7, 8, we identified the chromosomal regions harboring these genes in reedfish and searched in these blocks for neighboring genes that belonged to families with at least three members (see section 3.4.5). For the neighboring gene families, phylogenetic trees in ENSEMBL and Panther were inspected and those that seemed to have expanded in the same time period as the basal vertebrate genome duplications, phylogenetic trees were calculated. References and location of TRPV paralogon neighboring genes families are provided in **Supplementary Table S3**.

## 3 Results and discussion

As compared to previous studies, our present analysis includes more vertebrate species at key phylogenetic positions as well as sequences from representatives of major non-vertebrate metazoan groups. This allowed us to explore in more detail the relationships between non-vertebrate and vertebrate TRPVs. In the vertebrates, we have consistently compared sequence-based phylogenetic data with chromosomal locations of the genes in the many high-quality genome assemblies in order to explore conservation of synteny. In addition, we have compared the chromosomal neighborhood to investigate whether gene duplicates located on separate chromosomes within a species can be explained by duplications of chromosomal regions that coincide with the 1R, 2R, 3R and 4R whole-genome duplications (WGDs). All these types of information are important for the classification of the genes as orthologs or paralogs, and hence for assignment of gene names. In vertebrates, we have revealed three new TRPV types that arose in the early stages of vertebrate evolution, and we have named these TRPV7, TRPV8 and TRPV9. We have also found numerous independent gains and losses of TRPV family members in various vertebrate lineages and species.

### 3.1 TRPV gene searches identified new TRPV sequences

Annotated TRPV sequences were retrieved using BLAST searches in the NCBI or Ensembl genome databases. The TBLASTN results also revealed the existence of many non-annotated TRPV sequences that could be included in this study (see **Supplementary Table S1**), such as two TRPVA and B sequences in an ascidian, *C. savignyi;* paralogs of TRPV1 in some teleosts; paralogs of TRPV3 in coelacanth; paralogs of TRPV5 in green anole, Leishan spiny toad and painted turtle; and paralogs of TRPV7 in reedfish and platypus (see **Supplementary Table S1**). Coelacanth TRPV1, located on two distinct scaffolds, was manually curated and partial sequences of coelacanth TRPV3 and platypus TRPV8 were manually completed.

BLAST searches in sauropsid genomes for TRPV5 paralogs led to the identification and manual extension and annotation of a pseudogene in seven bird species. Similarly, BLAST searches in metatherian genomes led to the identification and manual assembly of a TRPV8 pseudogene in the gray short-tailed opossum (*Monodelphis domestica*). Sequences of bird TRPV5 and opossum TRPV8 pseudogenes were assembled using the CLC Main Workbench software and have been included in the phylogenetic analyses of the TRPV5-8 subfamily. Gene names were assigned by phylogenetic analyses and confirmed by conserved syntenies.

BLAST analyses allowed us to retrieve two TRPV genes with completely different names, rainbow trout ECaC (epithelial calcium channel) and *Xenopus* CAT1 (calcium transporter 1). Phylogenetic analyses permitted us to assign both ECaC and CAT1 to the TRPV family, therefore we propose renaming them as orthologs of TRPV5 (ECaC=TRPV5β and CAT1=amTRPV5-4). The corresponding genes in rat were likewise renamed (42). In the annelid the vent worm, we could identify as TRPVC/D the “hypothetical protein LSAT2”.

### 3.2 Global phylogeny of metazoan TRPV reveals previously undescribed TRPV types

To analyze the TRPV sequences in metazoans, we performed a phylogenetic analysis of 160 TRPV amino acid sequences including 40 sequences from non-vertebrates and 120 sequences from vertebrates; the tree also included a few (15) non-TRPV (TRPA, TRPM, TRPN and TRPP) sequences from vertebrates and non-vertebrates and was rooted with fruit fly and mouse TRPP sequences.

**Figure 1:**
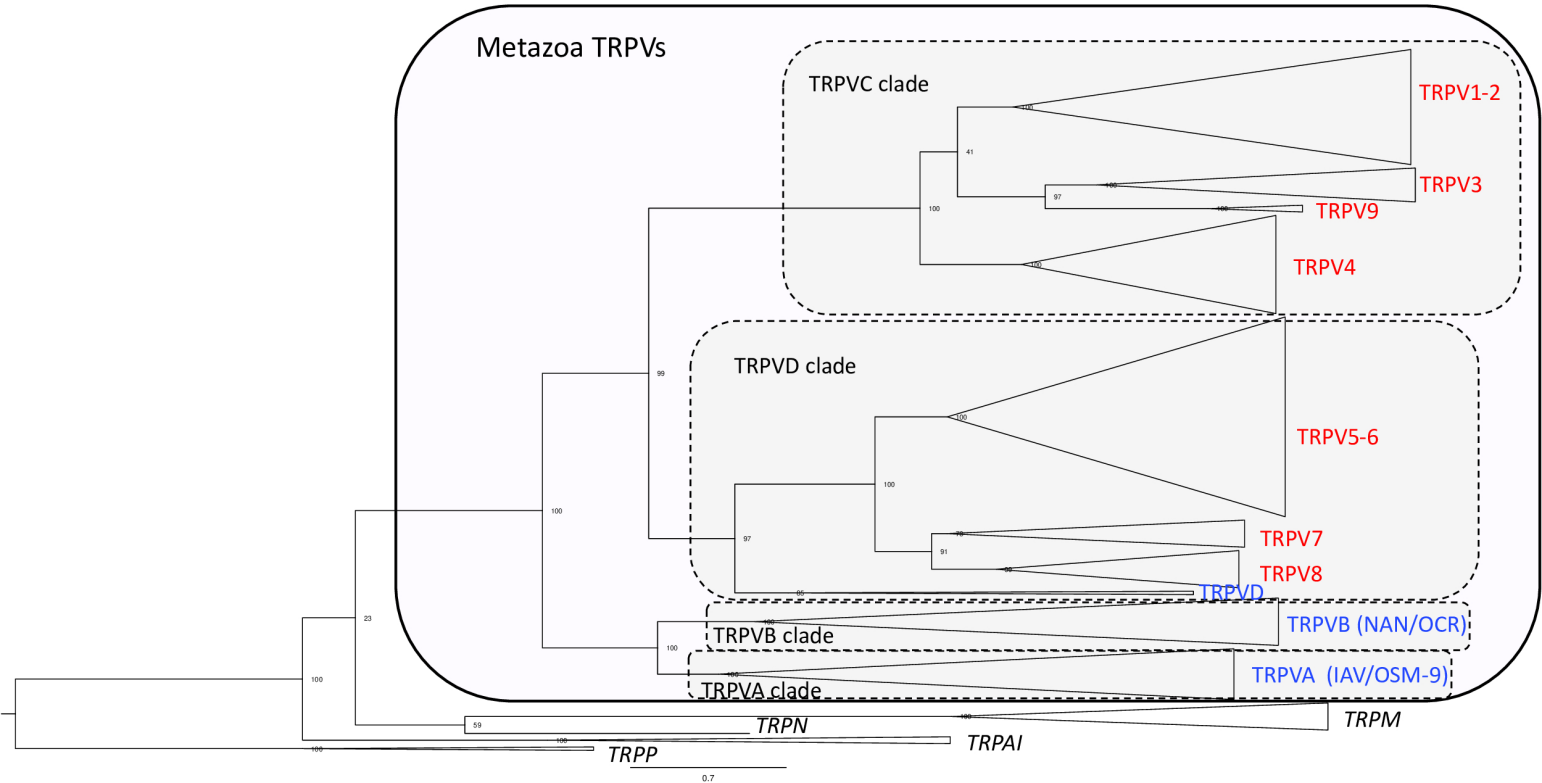
Global phylogenetic relationships of metazoan TRPV sequences. Tree topology inferred with the phylogenetic maximum likelihood method from an alignment of 160 TRPV amino acid sequences including 40 sequences from non-vertebrates and 120 sequences from vertebrates; the tree also included 15 TRP non-TRPV (TRPA, TRPM, TRPN and TRPP) sequences from vertebrates and non-vertebrates and was rooted with fruit fly and mice TRPP sequences. Boostrap values over 1000 replicates (%) are indicated. This global phylogenetic analysis clusters metazoan TRPV sequences into four major clades TRPVA, B, C and D. Vertebrate TRPV sequences are in clades C and D. Vertebrate TRPV sequences cluster in seven major clades (TRPV1-2; TRPV3; TRPV4, TRPV5/6, TRPV7, TRPV8, TRPV9) revealing three novel TRPV types, TRPV7, TRPV8 and TRPV9. See **Supplementary Figure S1** for detailed **Figure 1** with all individual sequences represented. See **Table S1** for sequences accession numbers.

In the phylogenetic tree (**Figure 1** and **Supplementary Figure S1** and **Table S1** for detailed information), all the metazoan sequences that have been classified in previous studies as TRPV were found to form a single large clade with the TRPA/TRPN sequences as the closest sister group, which is compatible with previous studies (3, 30, 43). The metazoan TRPV clade displays four main well-supported sub-clades (between 96%- and 100%-ML bootstrap probability values), that we have named TRPVA, B, C and D (**Figure 1**). On the one hand, TRPVA and B clades are sister clades and on the other hand, TRPVC and D clades are sister clades, suggesting that they resulted from duplication of ancestral genes TRPVA/B and TRPVC/D, respectively, which in turn came from a single ancestral TRPV gene. These hypotheses on the early evolutionary scenario of metazoan TRPV are shown on **Supplementary Figure S2A**.

Ecdysozoan TRPV sequences, fruit fly IAV and *C. elegans* OSM-9, clustered in clade TRPVA and fruit fly NAN and *C. elegans* OCR clustered in clade TRPVB (**Figure 1** and **Supplementary Figure S1**). Nematode-specific serial gene duplications likely gave rise to their four TRPVB paralogs, corresponding to OCR-1 to OCR-4, as previously reported (28). TRPVA and B clades encompass only non-vertebrate TRPV sequences, including both non-bilaterians (cnidarians: corals) and bilaterians, in agreement with 3. Concerning lophotrochozoans, we found that TRPV sequences from annelids (vent worm) and mollusks (great scallop), previously annotated as TRPV5-like, 6 or 6-like, clustered in TRPVA and TRPVB clades, allowing us to rename them TRPVA and TRPVB (**Supplementary Figure S1** and **Table S1**). Two TRPVA sequences were retrieved in the great scallop, indicating a mollusk or species-specific duplication (**Supplementary Figure S2A**). Furthermore, we also found that some deuterostome TRPV sequences clustered in TRPVA and B clades. This is the case for representative species of ambulacraria (hemichordates and echinoderms), and chordates (cephalochordates and urochordates). These sequences were either non annotated (*C. savignyi*) or annotated as TRPV, TRPV5, TRPV6, TRPV5-like or TRPV6-like, and we have named them TRPVA or TRPVB. We found two TRPVA sequences in hemichordates and echinoderms, and the phylogenetic analyses suggested that they resulted from independent gene duplications in each of these two lineages. Our data suggest that TRPVA and B genes were lost in deuterostomes after the split between urochordates and vertebrates.

The TRPVC/D clade encompasses at its base sequences from lophotrochozoans (annelid and mollusk). This is the first evidence of this TRPV type in protostomes. These sequences were previously annotated TRPV6, TRPV5-like or hypothetical protein LSAT2 (**Figure 1**, **Supplementary Figure S1** and **Table S1**) and we have named them TRPVC/D. These sequences may reflect the conservation in extant lophotrochozoans of an ancestral TRPVC/D gene (**Supplementary Figure S2A**). TRPVC and TRPVD are sister clades (**Figure 1**, **Supplementary Figure S1** and **Table S1**). A parsimonious hypothesis is that a duplication of TRPVC/D gene into TRPVC and TRPVD genes may have occured in an ancestral deuterostome (**Supplementary Figure S2A**).

The TRPVC clade includes only vertebrate sequences, suggesting the loss of this gene in non-vertebrate deuterostomes (**Supplementary Figure S2A**). Vertebrate TRPVC sequences clustered into four clades (TRPV1-2, TRPV3, TRPV9, TRPV4) (**Figure 1**), indicating, as for TRPVD, multiple duplications of TRPVC in vertebrates. This revealed three undescribed vertebrate TRPV types: TRPV7, 8 and 9. Further phylogenetic and synteny analyses of the vertebrate TRPVC and TRPVD subfamilies are provided in the following sections.

The TRPVD clade includes sequences from both vertebrates and non-vertebrate deuterostomes (hemichordates and cephalochordates). To the best of our knowledge, this is the first report of this third type of TRPV (in addition to TRPVA and B) in hemichordates and cephalochordates. We have renamed as TRPVD these non-vertebrate deuterostome sequences previously annotated as TRPV6-like. TRPVD sequences from non-vertebrate deuterostomes clustered basally to all vertebrate TRPVD sequences in agreement with species phylogenetic relationships (**Figure 1** and **Supplementary Figure S1**). We could find no TRPVD sequences in the other non-vertebrate species investigated (cnidarians, protostomes, echinoderms, urochordates). Following the parsimonious hypothesis that TRPVD gene arose from a duplication of TRPVC/D gene in ancestral deuterostome, this suggests independent losses of TRPVD in echinoderms and urochordates (**Supplementary Figure S2A**). TRPVD clade encompassed three vertebrate TRPV clades (TRPV5/6, TRPV7 and TRPV8) (**Figure 1**), indicating duplications of TRPVD in vertebrates.

Taken together, the early divergence of the A/B and C/D clades suggests that both of these existed in early bilaterians before the divergence of protostomes and deuterostomes, and most likely even earlier, before the divergence of Cnidaria and Bilateria (**Figure 1** and **Supplementary Figure S2A**). This implies that there have been lineage-specific losses of TRPVA and TRPVB in the vertebrate ancestor and TRPVC/D in Cnidaria and Ecdysozoa (**Supplementary Figure S2A**).

### 3.3 Investigation of vertebrate TRPV1, 2, 3, 4, 9 subfamily (clade TRPVC)

**Figure 2:**
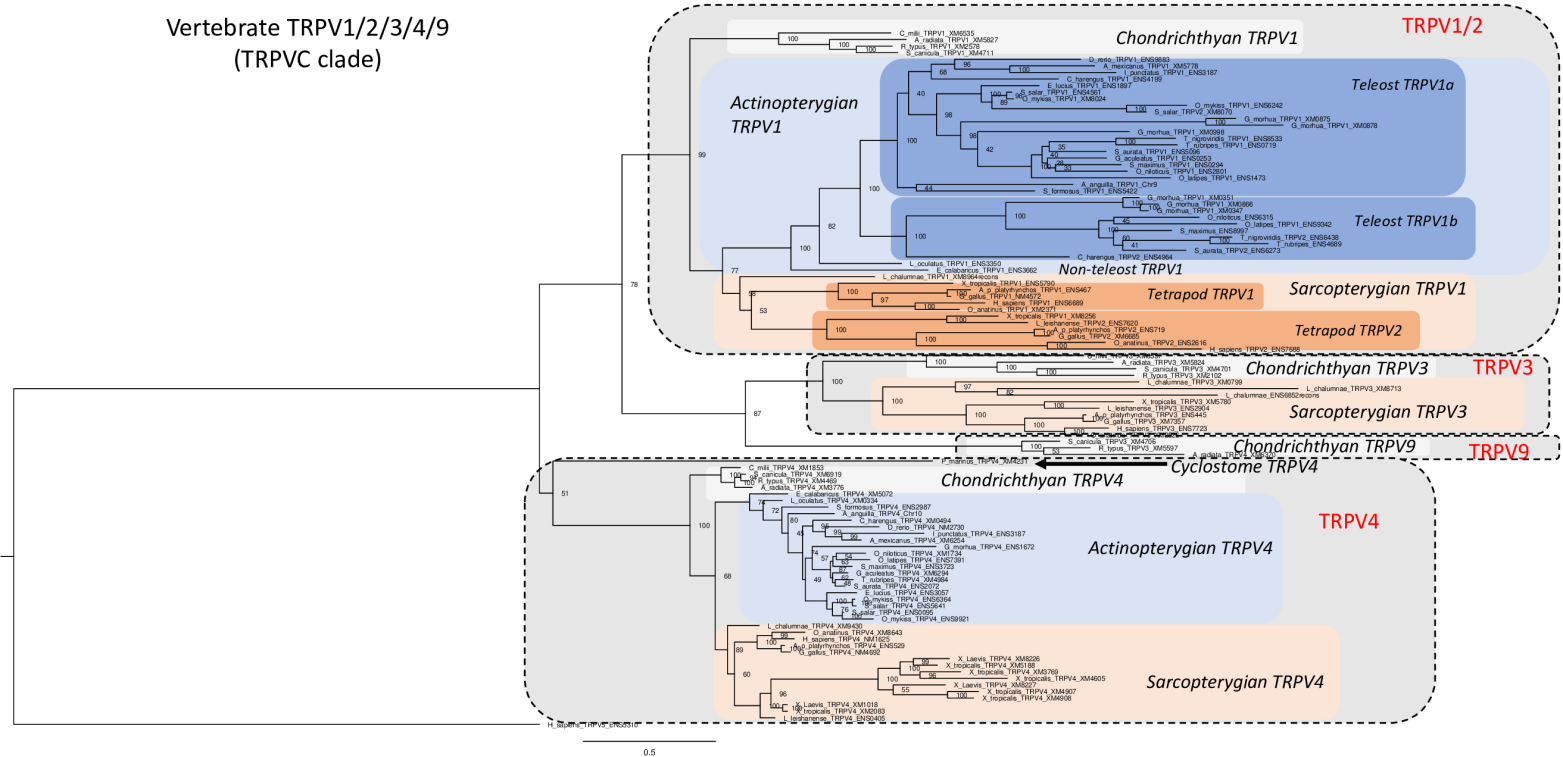
Phylogenetic relationships of vertebrate TRPV1,2,3,4,9 sequences (TRPVC). Tree topology inferred with the phylogenetic maximum likelihood method from an alignment of 105 amino acid sequences of vertebrate (cyclostome, chondrichthyan, actinopterygian and sarcopterygian) species, with human TRPV5 used to root the tree. Boostrap values over 1000 replicates (%) are indicated. See **Supplementary Table S1** for sequences accession numbers. This phylogenetic analysis clusters vertebrate TRPV1,2,3,4,9 sequences (TRPVC) into two main clades (TRPV1,2,3,9 and TRPV4). The TRPV1,2,3,9 clade encompasses two sister clades, TRPV1,2 clade and TRPV3,9 clade. Duplicated TRPV1a and b paralogs are found in some teleosts. TRPV2 is specific of tetrapods as sister clade of TRPV1. The novel TRPV9 clade, revealed by the present study, is the sister clade of TRPV3. TRPV3 is lacking in actinopterygians. TRPV9 is conserved only in chondrichthyans. Serial duplications of TRPV4 are specific of *Xenopus*.

We have investigated in total 105 vertebrate TRPV1, 2, 3, 4, 9 amino acid sequences, including one partial sequence from a cyclostome, 15 sequences from chondrichthyans, 53 from actinopterygians, and 36 sequences from sarcopterygians. The phylogenetic tree in **Figure 2** was rooted with the human TRPV5 sequence (for sequence references see **Supplementary Table S1**). In agreement with **Figure 1**, vertebrate TRPV1, 2, 3, 4, 9 sequences form two main clades, one containing TRPV1, 2, 3, 9 while the other consists only of orthologs of TRPV4 (as confirmed by conserved synteny in the species that have been investigated with regard to chromosomal location, see synteny analyses sections below).

#### 3.3.1 Phylogeny of TRPV1, 2, 3, 9 sequences

The 1, 2, 3, 9 group consists of four major clades and three of these (TRPV1/3/9) diverged from each other before the radiation of gnathostomes (chondrichthyans, actinopterygians and sarcopterygians) (**Figure 2**). These genes are located on the same chromosome in various vertebrate species (see synteny analyses, sections 3.3.2 and 3.3.5) and most likely arose by local duplications. The subtype named TRPV9 has only chondrichthyan sequences, but as it diverges from TRPV3 early in the tree in **Figure 2**, before TRPV3 displays its gnathostome radiation, it seems that TRPV9 is an ancient gnathostome TRPV type that was lost in osteichthyans. The subtype TRPV2 is found only in amphibians, mammals and sauropsids and likely arose by local duplication in the ancestor of tetrapods (see synteny analyses, sections 3.3.2 and 3.3.5)

##### 3.3.1.1 *Phylogeny of* TRPV1

TRPV1 is present in all gnathostomes investigated but could not be found in the genome assembly of a cyclostome, the sea lamprey (**Figure 2**), which suggests a loss of TRPV1 in cyclostomes (**Supplementary Figure S2B**). However, considering the current status of the lamprey genome with small scaffolds, it is still difficult to definitely conclude if TRPV1 has been lost in cyclostomes or not. TRPV1 sequences of two non-teleost actinopterygians, spotted gar and reedfish, diverge basally to the teleost TRPV1 sequences in agreement with their established phylogenetic positions. Teleosts have two TRPV1 clades; the one named TRPV1a is present in all teleost species studied whereas TRPV1b is present in some teleosts, such as clupeiforms (Atlantic herring *Clupea harengus*), various acanthopterygian groups, perciforms (Nile tilapia *Oreochromis niloticus* and gilthead seabream *Sparus aurata*), tetraodontiforms (fugu *Takifugu rubripes* and tetraodon *Tetraodon nigroviridis*), beloniforms (Japanese medaka *Oryzias latipes*), and pleuronectiforms (turbot *Scophthalmus maximus*). The presence of two TRPV1 paralogs in some teleost species has been previously reported (Fugu, tetraodon: 20; tilapia, codfish: 44).

The two TRPV1 paralogs in teleosts likely resulted from the teleost WGD (3R) event, as suggested by Saito etal. (20). This is supported by the similarities between the two TRPV1 chromosomal region in medaka, zebrafish, European eel, Atlantic cod and northern pike (see section 3.3.2.). While some teleosts have retained both of the resulting TRPV1 3R paralogs, independent TRPV1 gene losses have occurred in various lineage/species such as in elopomorphs (European eel), osteoglossomorphs (Asian bonytongue), siluriforms (channel catfish *Ictalurus punctatus)*, cypriniforms (Mexican tetra *Astyanax mexicanus*, zebrafish *Danio rerio*), and some acanthopterygian gasterosteiforms (stickleback *Gasterosteus aculeatus*). Thus, there is considerable species/lineage variation in the retention or loss of duplicated TRPV1. Furthermore, in the Atlantic cod (*Gadus morhua*), BLAST search revealed six TRPV1 sequences and our phylogenetic analysis suggests that independent serial local duplications led to three TRPV1a and three TRPV1b copies. Concerning salmonids, our study revealed the presence of duplicated TRPV1 in *Salmo* (Atlantic salmon, *Salmo salar*) and *Oncorhynchus* (rainbow trout) genus; these TRPV1 paralogs clustered together with the single northern pike (*Esox Lucius*) TRPV1a, which may suggest that the two salmonid TRPV1 paralogs resulted from the salmonid-specific WGD (4R). Further synteny analysis has been performed to test these hypotheses (see section 3.3.2.).

##### 3.3.1.2 Phylogeny of TRPV2

BLAST searches revealed that TRPV2 is only present in tetrapods (**Figure 2**). In our phylogenetic tree, tetrapod TRPV2 is a sister clade of tetrapod TRPV1 with the single coelacanth TRPV1 branching basally to both tetrapod TRPV1 and TRPV2. This suggests that TRPV2 likely results of a local gene-specific duplication of TRPV1 in the tetrapod lineage. However, this conclusion is tentative because the phylogenetic position of coelacanth sequences may not always be correct due to low evolutionary rate.

##### 3.3.1.3 Phylogeny of TRPV3

The TRPV3 gene is present in chondrichthyans (elephant shark, spotted catshark, whale shark and thorny skate) and sarcopterygians (from coelacanth to human) but absent in all actinopterygians investigated (Polypteridae, holostean and teleosts) (**Figure 2**). The TRPV3 clade constitutes a sister clade of the TRPV1, 2 clade. Saito etal. (20) suggested that TRPV3 was produced by gene duplication of TRPV1 in a common ancestor of teleosts and terrestrial vertebrates, and that TRPV3 would have been lost in the teleost fishes. Our study includes a number of chondrichthyans and supports an early origin of TRPV3 before the gnathostome radiation and its loss in the actinopterygian ancestor even before the divergence of Polypteridae. This is further supported by the synteny and paralogon analysis (sections 3.3.2. and 3.3.5.). The coelacanth genome presents three TRPV3 genes which cluster together in the phylogenetic tree (**Figure 2**), suggesting duplications in the actinistian lineage.

##### 3.3.1.4 Discovery and phylogeny of TRPV9

Our study revealed the existence of a previously undescribed vertebrate TRPV type that we have named TRPV9, which is only present in elasmobranchs (spotted catshark, whale shark and thorny skate) among extant vertebrates. As we could not find this gene in the elephant shark genome, it may have been lost in holocephalan lineage. The TRPV9 clade is a sister clade to the TRPV3 clade, which suggests that the gene duplication occurred in a gnathostome ancestor. As TRPV9 was not identified in any other gnathostomes, it was probably lost in the ancestor of the osteichthyan lineage (**Supplementary Figure S2B**).

#### 3.3.2 Conserved synteny of the 1, 2, 3, 9 genomic region

**Figure 3:**
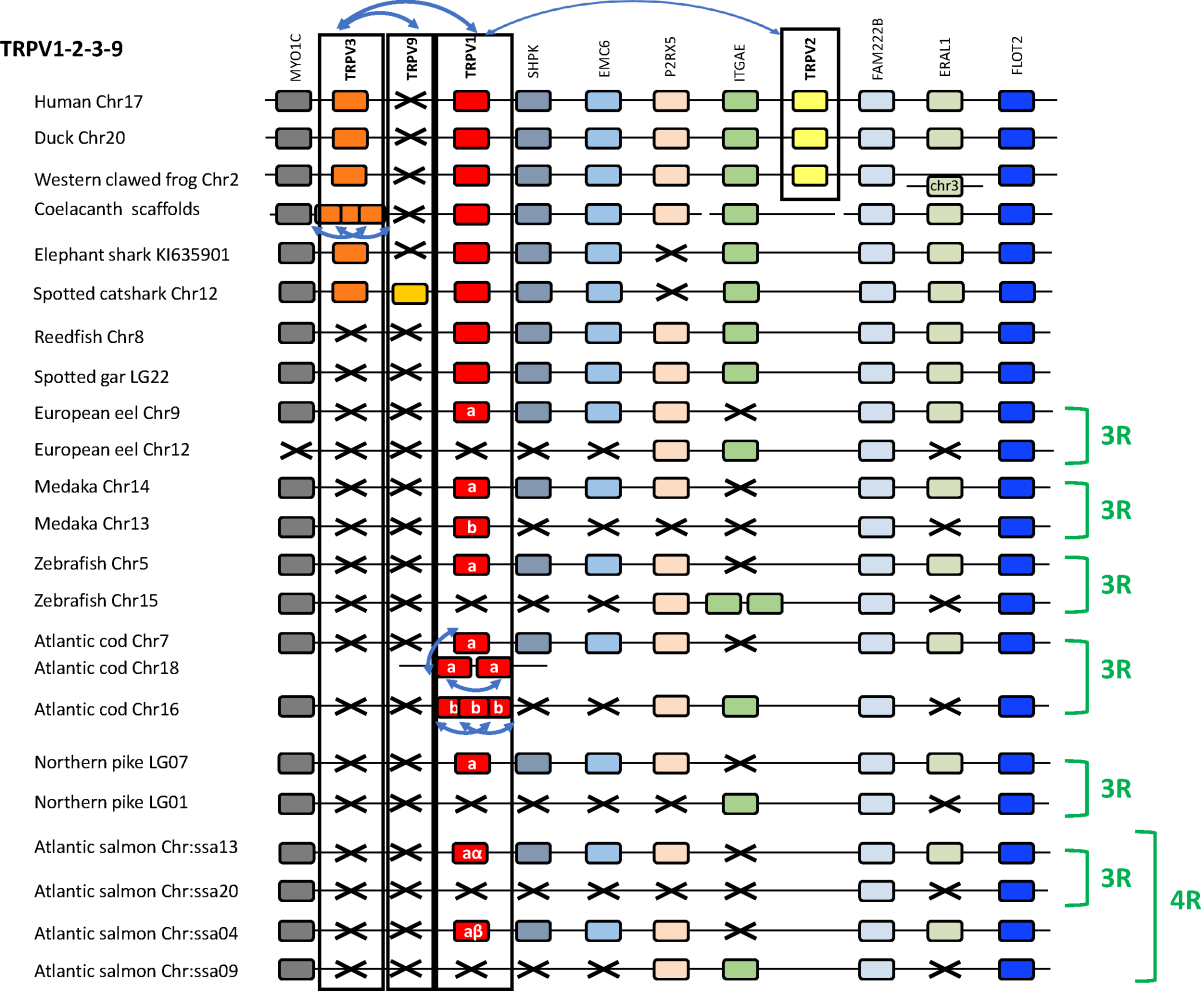
Conserved synteny between vertebrate TRPV1,2,3,9 genomic regions. Human TRPV1,2,3 genomic region is used as template. Eight neighboring genes are shown. Gene colors are applied in order to show conserved synteny as well as sequence homology between representative vertebrate species: sarcopterygians (mammal, sauropsid, amphibian, actinistian), chondrichthyans, actinopterygians (Polypteridae, holostean, teleosts). Black frames highlight orthologous TRPV genes between vertebrate species. Blue arrows indicate TRPV-specific local gene duplication and black cross, gene missing. TRPV1 and TRPV3 are in tandem position in osteichthyans, reflecting an ancient local duplication. TRPV3 has been lost in the actinopterygian lineage. TRPV2 located in the same genomic region as TRPV1 and TRPV3, is present only in tetrapods and likely results from a local gene duplication in this lineage. The novel type TRPV9 present in chondrichthyans is also located in the same genomic region between TRPV1 and TRPV3 likely reflecting an ancient local gene duplication. TRPV9 has been lost in the osteichtyan lineage. The genomic region has been duplicated *via* the teleost-specific whole genome duplication (3R) leading to duplicated paralogs of TRPV1 (TRPV1a and TRPV1b). TRPV1a has been conserved in all teleost, while TRPV1b has been lost repeatedly and independently in some teleosts such as the eel, zebrafish, northern pike. Both TRPV1a and TRPV1b paralogs have undergone serial gene duplication specifically in a gadiform, the cod. The genomic region has been further duplicated *via* the salmonid-specific whole genome duplication (4R) leading to duplicated paralogs of TRPV1a (TRPV1aα and TRPV1aβ). See **Supplementary Table S2** for TRPV and neighboring genes sequences accession numbers. Coelacanth scaffolds: JH127958/JH129978/JH129026/JH130358/JH129740/JH127350/.

The chromosomal locations of the 1, 2, 3, 9 genes are shown in **Figure 3** for a broad range of gnathostomes including several teleosts. All four genes are syntenic, consistent with origin by local duplications of TRPV1 (see also **Supplementary Table S2** and **Supplementary Figures S2B, C**). **Figure 3** displays four sarcopterygians (human, duck *Anas platyrhynchos platyrhynchos*, Western clawed frog and coelacanth), two chondrichthyans (elephant shark and spotted catshark), two non-teleost actinopterygians (reedfish and spotted gar), and six teleosts (Asian bonytongue, European eel, medaka, zebrafish, Atlantic cod, northern pike and Atlantic salmon). The human genomic region where TRPV1, TRPV2 and TRPV3 are located, was used as a template and eight neighboring genes were investigated: MYOC, SHPK, EMC6, P2RX5, ITGAE, FAM222B, ERAL1, FLOT2. Previously unidentified TRPV neighboring genes in some vertebrate genome databases were retrieved using the TBLASTN algorithm of the Ensembl Genome Browser website (see **Supplementary Table S2**). As shown in **Figure 3**, the genomic regions were conserved between human and all other vertebrate species investigated in this study. The TRPV9 gene present in elasmobranchs is also located in this genomic region.

The conservation of synteny confirms orthology of TRPV1 across chondrichthyans, sarcopterygians and actinopterygians. Some of the neighboring genes are duplicated in teleosts, which all display duplicated copies of MYOC (except the European eel), FAM222B and FLOT2. In contrast, the non-teleost actinopterygians, reedfish and spotted gar, possess only a single copy of these genes, supporting the hypothesis that the TRPV1 genomic region has been duplicated in the teleost ancestor as a result of 3R. The duplicated TRPV1 genes, named “a and b” according to Zfin nomenclature for teleost 3R-paralogs, have been retained in some but not all teleosts. The TRPV1 paralog conserved in all teleost species, named TRPV1a, was positioned in the same paralogon member for all teleosts (**Figure 3**). The other TRPV1 paralog, named TRPV1b, has been lost repeatedly in various teleosts. In the Atlantic cod, six TRPV1 paralogs were identified. The synteny analyses showed that the cod TRPV1 gene located on chromosome 7 is orthologous to TRPV1a in the other teleosts (**Figure 3**). Two other cod TRPV1 genes defined as “TRPV1a” by the phylogenetic analysis (**Figure 2**) correspond to species-specific serial gene duplications of TRPV1a, and translocation to chromosome 18. Three cod TRPV1 genes located on chromosome 16, and defined as “TRPV1b” by the phylogenetic analysis are co-orthologs of TRPV1b in other teleosts, and have resulted from species-specific serial gene duplications of TRPV1b. In the Atlantic salmon, four copies of MYOC, FAM222B and FLOT2 have been identified, reflecting that the genomic region of TRPV1 has been further duplicated as a result of the salmonid-specific 4R. The TRPV1a gene is retained in all teleosts including the Northern pike and inherited by the salmonid lineage where it was duplicated by 4R, leading to two TRPV1a paralogs; we named them TRPV1aα and TRPV1aβ, according to the “α/β”nomenclature for salmonid 4R-paralogs (45, 46). The lack of TRPV1b in an Esocidae, the Northern pike, suggests loss of the TRPV1b paralog in the common ancestor of Esocidae and Salmonidae (**Supplementary Figure S2D**). It is noteworthy that physiological investigations in salmonids (*O. mykiss*, 21; *S. salar*, 22) did not consider the presence of two TRPV1 paralogs resulting from 4R as revealed in the present study. Further studies may aim at comparing the respective functions of both paralogs in salmonids.

The TRPV2 gene has only been identified in tetrapods in the same genomic region as TRPV1 and probably arose by local duplication of TRPV1 in a tetrapod ancestor (**Supplementary Figure S2C**). According to our phylogenetic and synteny analyses, the tetrapod TRPV2 gene is not orthologous to the duplicated TRPV1 gene in teleosts, meaning that the gene annotated as “TRPV2” in herring, tetraodon and salmon in the Ensembl and/or NCBI databases has been incorrectly named and should be renamed TRPV1b.

TRPV3 was found to be syntenic with TRPV1 in human and other tetrapods in agreement with previous reports. Saito etal. (20) proposed that TRPV3 was generated by tandem gene duplication of TRPV1 in the common ancestor of teleosts and terrestrial vertebrates. Our study shows that TRPV3 was already present in the chondrichthyan ancestor and is syntenic with TRPV1 gene in holocephalan (elephant shark) and elasmobranch (spotted catshark) (**Figure 3**), pushing back the origin of the TRPV1/TRPV3 duplication to at least an ancestral gnathostome (**Supplementary Figure S2B**). In coelacanth, three TRPV3 paralogs were identified syntenic to TRPV1, indicating a species-specific serial duplication of TRPV3 in actinistians in agreement with the phylogenetic analysis. Synteny analysis further confirmed that TRPV3 is missing in the TRPV1 genomic region of all actinopterygians investigated, including polypterid, holostean and teleost species, supporting the loss of TRPV3 in the actinopterygian ancestor (**Supplementary Figure S2D**).

The TRPV9 gene identified in elasmobranchs is located between the TRPV1 and the TRPV3 genes (**Figure 3**). The phylogenetic analyses (**Figures 1**, **2**) indicate that TRPV9 is the sister clade to TRPV3 with a likely origin in a gnathostome ancestor. The synteny analysis supports the origin of TRPV3/TRPV9 by gene-specific local duplications. While TRPV3 has been kept in chondrichthyans and sarcopterygians, TRPV9 has been retained only in elasmobranchs.

#### 3.3.3 Phylogeny of TRPV4

As for TRPV1, the TRPV4 gene is present in all gnathostomes. The phylogenetic tree displays shorter branch lengths for TRPV4 than for 1, 2, 3, 9 sequences, suggesting a higher conservation of TRPV4 (**Figure 2**). The only exception is the additional TRPV4 duplicates in the genus *Xenopus*, but it is frequently observed that duplication leads to increased evolutionary rate. A partial sequence homologous to TRPV4 could be retrieved from the sea lamprey genome, indicating an early origin of TRPV4 in a vertebrate ancestor. Only a single TRPV4 gene was found in each of the actinopterygian non-teleost and teleost species studied, except for Atlantic salmon and rainbow trout, which exhibited two paralogs. This may indicate that 3R did not impact the number of TRPV4 in teleosts, suggesting a post-3R loss of one of the teleost-specific duplicated TRPV4 genes, before the emergence of elopomorphs. The two TRPV4 genes in Atlantic salmon and rainbow trout may have resulted from the salmonid-specific genome duplication (4R), a hypothesis that was further assessed by synteny analyses.

All chondrichthyan and sarcopterygian species possess a single TRPV4 gene, except two anuran species from the genus *Xenopus*. The Western clawed frog exhibited six TRPV4 genes, and the African clawed frog (*Xenopus laevis*) three TRPV4 genes. Multiple TRPV4 genes were previously reported in the Western clawed frog (47). In the present study we found that another anuran, the Leishan spiny toad (*Leptobrachium leishanense*), displayed only one TRPV4 gene, which allows us to hypothesize that the serial duplications of TRPV4 genes occurred specifically in the *Xenopus* genus (**Supplementary Figure S2C**). While one of the *Xenopus* TRPV4 sequences has a short branch, the other sequences all have long branches indicating rapid divergence after the gene duplications (**Figure 2**).

#### 3.3.4 Conserved synteny of the TRPV4 genomic region

**Figure 4:**
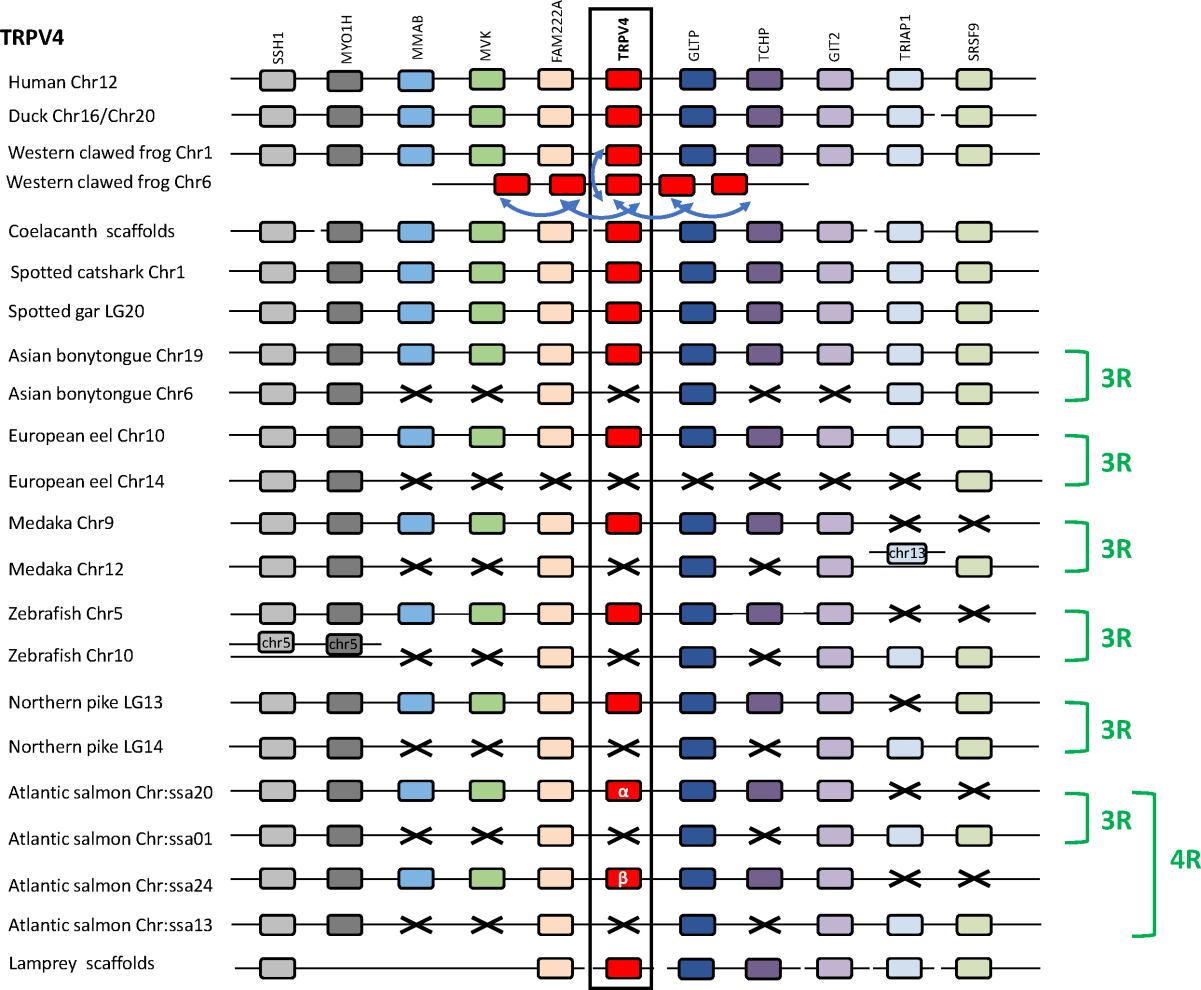
Conserved synteny between vertebrate TRPV4 genomic regions. Human TRPV4 genomic region is used as template. Ten neighboring genes are shown. Gene colors are applied in order to show conserved synteny as well as sequence homology between representative vertebrate species: cyclostome, chondrichthyans, sarcopterygians (mammal, sauropsid, amphibian, actinistian), actinopterygians (holostean, teleosts). Black frame highlights orthologous TRPV genes between vertebrate species. Blue arrows indicate TRPV-specific local gene duplication and black cross, gene missing. In amphibians multiple additional TRPV4 paralogs, translocated on another non-homologous genomic region, result from serial gene duplications (blue arrow). The TRPV4 genomic region has been duplicated *via* the teleost-specific whole genome duplication (3R) but a single TRPV4 paralog is present in extant teleosts, suggesting an early loss of one paralog after the 3R. The genomic region has been further duplicated *via* the salmonid-specific whole genome duplication (4R) leading to duplicated paralogs of TRPV4 (TRPV4α and TRPV4β). See **Supplementary Table S2** for TRPV and neighboring genes sequences accession numbers. Coelacanth scaffolds: JH127860/JH127626/JH127089/JH126570. Sea lamprey scaffolds: Chr 5/Unplaced Scaffold/77 Unlocalized Scaffold/Chr 74/84 Unlocalized Scaffold.

Analysis of TRPV4 neighboring genes was performed in four sarcopterygians (human, duck, Western clawed frog and coelacanth), a chondrichthyan (spotted catshark), seven actinopterygians (spotted gar, Asian bonytongue, European eel, medaka, zebrafish, northern pike and Atlantic salmon), and a cyclostome, the sea lamprey (**Figure 4**). The human TRPV4 genomic region was used as template and ten TRPV4 neighboring genes were investigated: SSH1, MYO1H, MMAB, MVK, FAM222A, GLTP, TCHP, GIT2, TRIAP1, SRSF9, all of which were found to be located in the TRPV4 genomic region of the other gnathostomes studied. In the sea lamprey, some neighboring genes could be also retrieved in the small scaffolds available.

In the Western clawed frog *Xenopus tropicalis*, one TRPV4 gene has been identified on chromosome 1, indicating orthology with TRPV4 in other vertebrates. The five additional TRPV4 genes have been identified on *X. tropicalis* chromosome 6, presumably as a result of translocation and serial gene duplications. A similar result was found in the *X. laevis* genome, where one TRPV4 gene was located on chromosome 1 and was orthologous to TRPV4 in other vertebrates, while two other TRPV4 genes were located on chromosome 6 (not shown). In the Leishan spiny toad, only a single TRPV4 gene has been identified on chromosome 1 (not shown). These results complement our phylogenetic analysis. Noticeably, the TRPV4 gene located on chromosome 1 in these anuran species, orthologous to other vertebrate single TRPV4, is the one presenting a short branch in the phylogeny (**Figure 2**) described above.

In teleosts, some of TRPV4 neighboring genes are present as duplicated paralogs in most species studied: SSH1, MYO1H, FAM222A, GLTP, GIT2. In contrast, the spotted gar possesses only a single copy of these genes, supporting a duplication of the TRPV4 genomic region in teleosts. The time frame for these duplications, and their relation to the species divergencies, is consistent with duplication as a result of teleost 3R. For some other neighboring genes of TRPV4, only a single paralog gene has been identified in all teleosts investigated in this study, including in basal teleosts (European eel and Asian bonytongue): MMAB, MVK, TRPV4 and TCHP. This is the case also for TRPV4 itself for which a single paralog has been conserved in all teleosts studied. The single TRPV4 gene has been conserved in the same paralogon member as MMAB, MVK, TRPV4 and TCHP in all teleost species. This suggests that the other TRPV4 paralog was lost shortly after 3R. In the Atlantic salmon, four copies of the TRPV4 neighboring genes SSH1, MYO1H, FAM222A, were identified, in agreement with the further duplication of the TRPV4 genomic region as a result of the salmonid-specific 4R WGD event. The single TRPV4 paralog, inherited from the teleost lineage, has been duplicated in salmonids by the 4R, leading to two TRPV4 paralogs which we propose to name TRPV4α and TRPV4β in agreement with the nomenclature of salmonid 4R-duplicates.

#### 3.3.5 Paralogon for vertebrate TRPV1,2,3,4,9 genes

**Figure 5:**
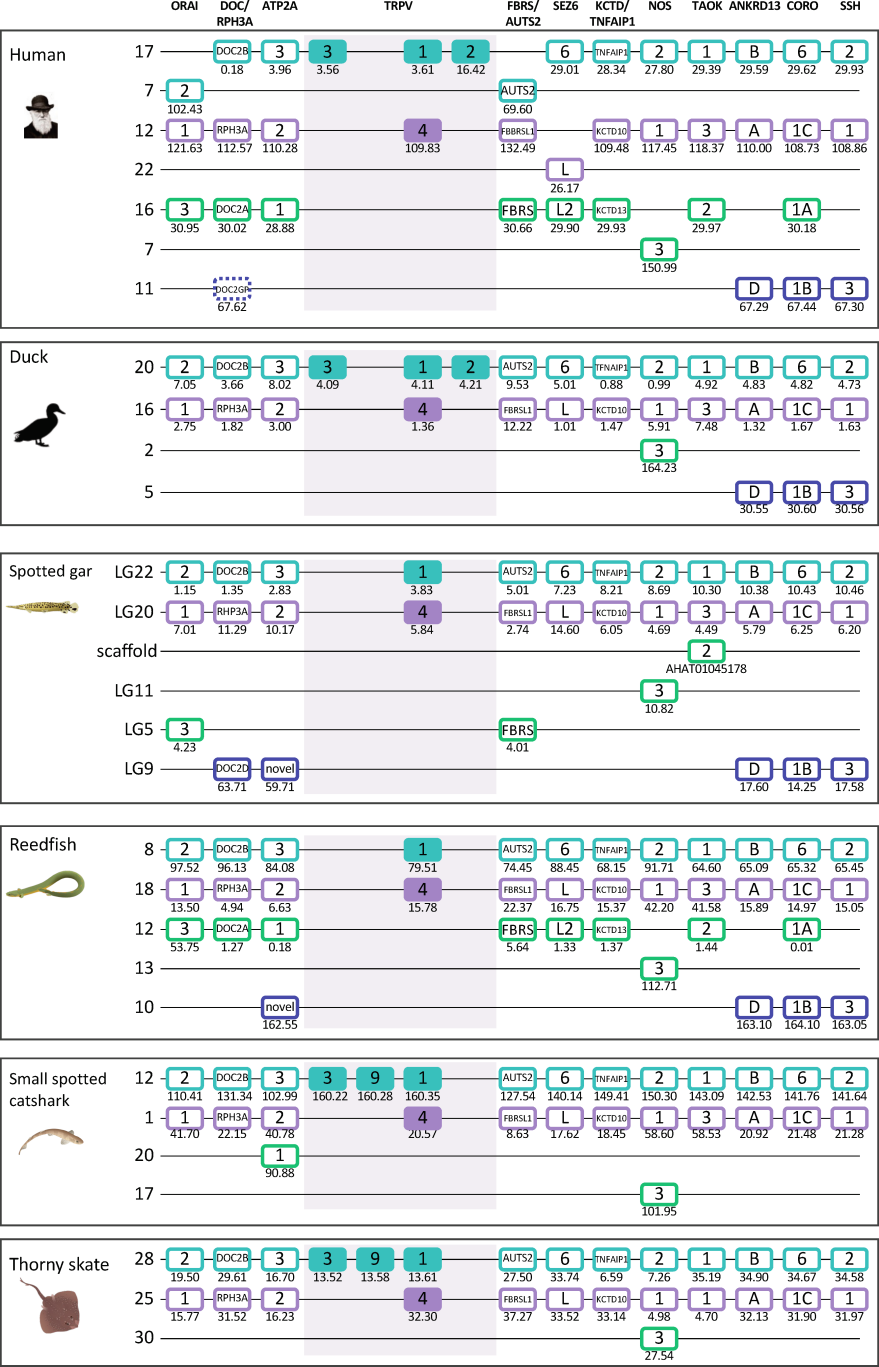
Paralogon for vertebrate TRPV1, 2, 3, 4, 9 genes. Gnathostome chromosomal regions harboring the genes of the TRPVC subfamily: the TRPV genes 1, 2, 3 and 9 are in a chromosomal region with several neighboring genes that have related genes also on the chromosome where TRPV4 is located, as well as two additional chromosomes which lack TRPV genes. Sequence-based phylogenetic analyses (**Supplementary Figure S3**) show that all of these gene families underwent duplications in time period of the WGD events 1R and 2R. Thus, these chromosomal regions are in agreement with quadruplication of an ancestral gnathostome (or vertebrate) chromosome. The species shown are human, duck (*Anas platyrhynchos*), spotted gar (*Lepisosteus oculatus*), reedfish (*Erpetoichthys calabaricus*), small spotted catshark (*Scyliorhinus canicula*), and thorny skate (*Amblyraja radiata*). The number below each gene shows the position in the chromosome. The order of the genes along the chromosomes has been re-shuffled to highlight the similarities. Animal illustrations are used with permission from Daniel Ocampo Daza, source: www.egosumdaniel.se, except the human image which is used with permission from https://commons.wikimedia.org and the duck image which is from http://phylopic.org.

Interestingly, the two main clades of TRPVC sequences, TRPV1, 2, 3, 9 and TRPV4, which are present on two separate chromosomes in all species investigated, share two neighbors that represent two other gene families; both chromosomes have members of the MYO1 and FAM222 families (**Figures 3**, **4**). This finding triggered us to search for additional neighboring gene families with representatives on these two chromosomes. Indeed, we found three neighboring gene families with a full quartet of members and as many as eight adjacent gene families with three members (**Figure 5** and section 3.5) indicating duplications of an ancestral chromosomal region in the two basal gnathostome WGD events, thus quadruplication resulting in a tetraparalogon (**Figure 5**).

Taken together, this pattern of chromosome similarities strongly suggests that TRPV1 and TRPV4 are duplicates that appeared as a result of a duplication of a large block of genes in the same time frame as the 1R/2R WGD events. TRPV3 and 9 arose in the gnathostome ancestor by local duplications of TRPV1. The TRPV2 gene appeared considerably later, in the tetrapod ancestor, as yet another copy of TRPV1. Further discussion on vertebrate TRPV paralogon is provided in section 3.5.

### 3.4 Investigation of vertebrate TRPV5/6, 7, 8 subfamily (clade TRPVD)

**Figure 6:**
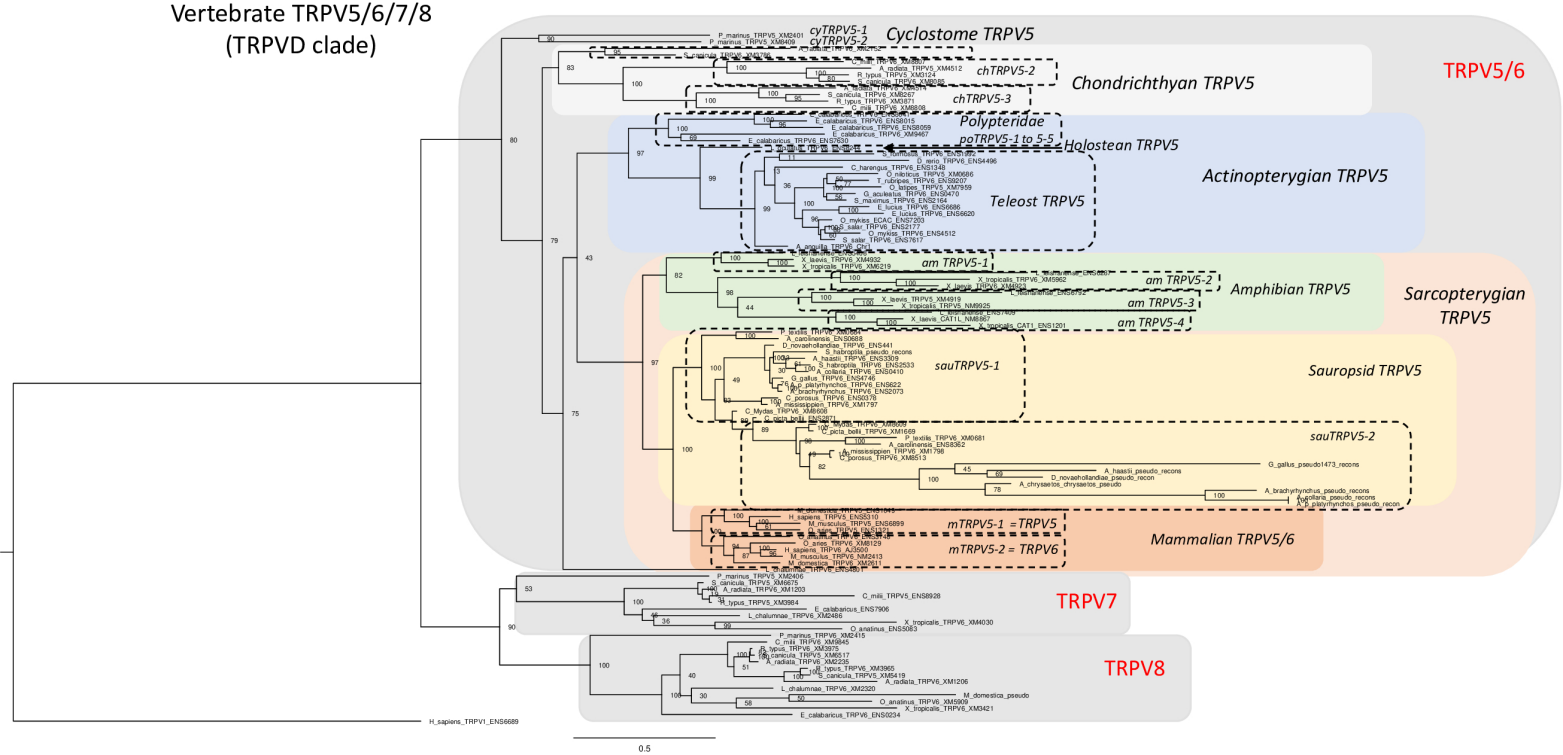
Phylogenetic relationships of vertebrate TRPV5/6, 7, 8 sequences (TRPVD). Tree topology inferred with the phylogenetic maximum likelihood method from an alignment of 105 amino acid sequences of vertebrate (cyclostome, chondrichthyan, actinopterygian and sarcopterygian) species, with human TRPV1 used to root the tree. Boostrap values over 1000 replicates (%) are indicated. See **Supplementary Table S1** for sequences accession numbers. This phylogenetic analysis clusters vertebrate TRPV5/6, 7, 8 (TRPVD) sequences into two main clades (TRPV5/6 and TRPV7, 8). TRPV5 has been duplicated repeatedly and independently in the various vertebrate lineages: in cyclostomes, chondrichthyans, amphibians, sauropsids, mammals (referred to as TRPV5 and TRPV6), Polypteridae and Esocidae. Among sauropsids, in birds, one of the TRPV5 duplicated paralogs has become a pseudogene. The TRPV7, 8 clade encompasses the two vertebrate TRPV types, TRPV7 and TRPV8, revealed by the present study. TRPV7 and TRPV8 are present in extant vertebrate representatives, from cyclostomes to prototherian mammals, and have been lost independently in neopterygian actinopterygians and in therian mammals. Duplicated paralogs of TRPV8 are present in elasmobranchs.

Our phylogenetic analysis of the TRPV5/6, 7, 8 subfamily includes 105 vertebrate sequences including 4 from a cyclostome, the sea lamprey, 21 from chondrichthyans, 23 from actinopterygians and 56 from sarcopterygians. The tree in **Figure 6** was rooted with the human TRPV1 sequence (for sequence references see **Supplementary Table S1**). As in the global phylogeny described in section 3.2 and **Figure 1**, the detailed phylogenetic tree of vertebrate TRPV5/6, 7, 8 splits the sequences into two main clades, one encompassing TRPV5/6 sequences and its sister clade encompassing the sequences that we have named TRPV7 and TRPV8. To the best of our knowledge, this is the first time the two latter subtypes are described, probably because neither exists in placental mammals.

The TRPV5/6 clade contains lineage-specific local duplications in as many as seven vertebrate lineages among those investigated in the present study. It is one of these lineage-specific duplicates, the one found in mammals, that has been previously named TRPV6. Hence, TRPV6 does not exist in any lineage that diverged earlier from the one leading to mammals. Orthology of TRPV5 is strongly supported by conserved synteny as described below. The TRPV7 and TRPV8, are sister clades, and TRPV7 and 8 genes are syntenic with each other in the species that have both of these genes (see synteny analyses section 3.4.4), suggesting that they arose from a local gene duplication.

#### 3.4.1 Phylogeny of TRPV5 and its duplicates, including TRPV6

TRPV5 is present in all gnathostomes included in our analyses. The phylogenetic tree in **Figure 6** is quite complex and suggests numerous lineage-specific duplication events. As pointed out above, the gene that has been named TRPV6 resulted from a duplication that took place in the mammalian lineage before the divergence of the eutherian and metatherian lineages, as also suggested in previous studies (20, 42, 48). This duplication probably occurred even before the divergence of prototherians as suggested by the phylogenetic tree although only one gene was found in platypus (TRPV6). Interestingly, the therian TRPV5 and TRPV6 genes have equally short branches, indicating very similar and rather high conservative selection pressure. The mammal-specific origin of TRPV6 means that the duplicates of TRPV5 that have arisen in other vertebrate lineages should not be named TRPV6. To be clear, in the present study we also referred to mammalian TRPV5 and TRPV6 as mTRPV5-1 and mTRPV5-2 (m for mammalian) (**Figure 6**).

All chondrichthyan sequences clustered in a clade that diverged at the base of all other gnathostome sequences (osteichthyans) in agreement with the species phylogeny. Three TRPV5-like genes were found in thorny skate and spotted catshark and were named chTRPV5-1, chTRPV5-2 and chTRPV5-3 (ch for chondrichthyan), reflecting serial gene duplications specific to chondrichthyans. chTRPV5-1 could not be identified in whale shark nor in the holocephalan elephant shark, suggesting independent losses in these two lineages.

Only one TRPV5 gene was found in the coelacanth, diverging at the base of the tetrapod clade, in agreement with the actinistian phylogenetic position among sarcopterygians.

All amphibian sequences clustered in a sister clade of the amniote sequences. Among amphibians, the phylogenetic tree displays as many as four sister clades, as previously observed by 48. Each clade encompasses one sequence from three anuran species (Western clawed frog, African clawed frog and Leishan spiny toad), suggesting that the duplications took place in the ancestor of the anuran lineage. We have named these amphibian paralogous genes amTRPV5-1, amTRPV5-2, amTRPV5-3, amTRPV5-4.

All the sequences of sauropsids clustered in a single clade, sister to the mammalian clade. Squamates, chelonians, crocodilians and birds display two genes, clustering in two sister clades indicating gene duplication in their common sauropsid ancestor as previously suggested (20, 48). We have named these genes sauTRPV5-1 and sauTRPV5-2. The former of these is highly conserved in all species whereas sauTRPV5-2 is well conserved in all except birds which display extensive divergence as shown by the long branches in the tree (**Figure 6**), in fact even to the point that it has become a pseudogene. This pseudogene was detected by BLAST searches in bird genomes in the Ensembl database. While the number of exons is 15 for green anole sauTRPV5-2, as for chicken sauTRPV5-1, we could detect for bird sauTRPV5-2 only 9 exons in duck, 6 in golden eagle (*Aquila chrysaetos chrysaetos*) and emu (*Dromaius novaehollandiae*), 5 in chicken (*Gallus gallus*), 3 in great spotted kiwi (*Apteryx haastii*) and in yellow-billed parrot (*Amazona collaria*), and only 1 exon in kakapo (*Strigops habroptila)* and in pink-footed goose (*Anser brachyrhynchus*).

All actinopterygian sequences clustered together in a sister clade to the osteichthyan clade (**Figure 6**). Five TRPV5-like are present in representatives of a basal group of actinopterygians, Polypteridae (reedfish and bichir). They clustered in a clade diverging at the base of all neopterygian (holostean and teleost) sequences (**Figure 6**) indicating that the five paralogs originated from serial duplications is specific to Polypteridae. In the Ensembl database these genes are named TRPV6, TRPV6-like or TRPV5-like. We have named these paralogs poTRPV5-1 to poTRPV5-5 (po for Polypteridae). In contrast, a single gene is present in a representative of another non-teleost actinopterygian groups, a holostean, the spotted gar. The single TRPV5 sequence of the gar diverged at the base of the teleost clade, in agreement with the phylogenetic position of holosteans within the actinopterygians. In teleosts, only a single gene has been identified in most species investigated including representatives of early diverging groups (Elopomorpha and Osteoglossomorpha). This suggests that the assumed 3R duplicate was lost soon after 3R before the teleost radiation. A few of the teleost species investigated do have TRPV5 duplicates: the northern pike has a lineage-specific local duplicate (**Figure 6** and synteny analysis section 3.4.2.), and the Atlantic salmon has a duplicate that most likely resulted from the salmonid-specific WGD (4R), as for duplicated TRPV1 and TRPV4, and is supported by synteny analysis (see sections 3.3.2 and 3.3.4).

Altogether, our phylogenetic analysis supports independent duplications of TRPV5 in the respective ancestors of mammals, sauropsids, amphibians (anuran) and chondrichthyans, as previously suggested by 48. We extend here this finding to Polypteridae, northern pike and Salmonidae (by 4R) (**Supplementary Figures S2C, D**). Furthermore, we retrieved two genes in a cyclostome, the sea lamprey (that we named cyTRPV5-1 and cyTRPV5-2). Their sequences clustered together and diverged at the base of the gnathostome clade, in agreement with the vertebrate phylogeny (**Figure 6**). This suggests that a TRPV5 gene duplication also occurred independently in cyclostomes.

#### 3.4.2 Conserved synteny of the TRPV5/6 genomic region

**Figure 7:**
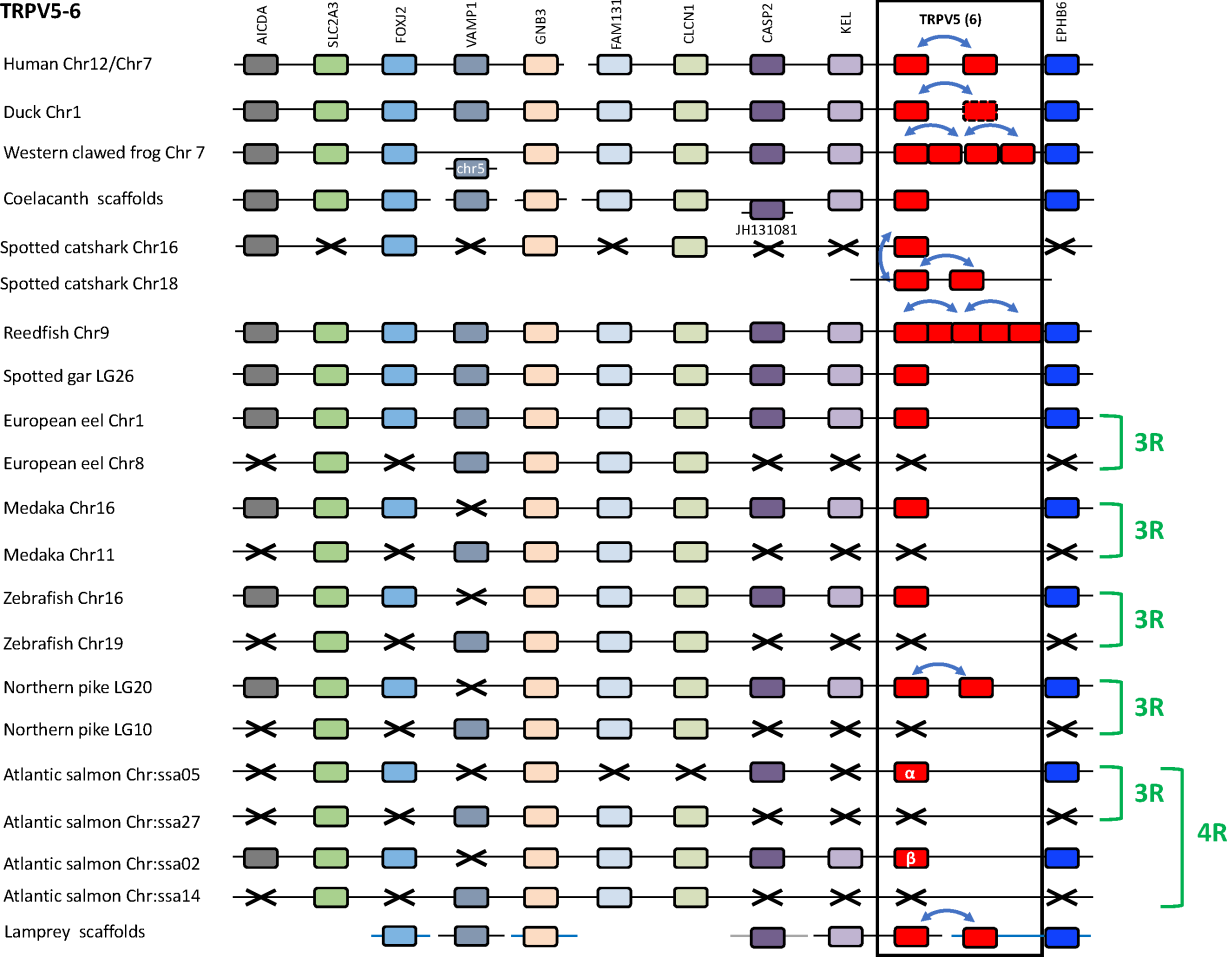
Conserved synteny between vertebrate TRPV5/6 genomic regions. Duck TRPV5 genomic region is used as template. Ten neighboring genes are shown. Gene colors are applied in order to show conserved synteny as well as sequence homology between representative vertebrate species: cyclostome, chondrichthyan, sarcopterygians (mammal, sauropsid, amphibian, actinistian), actinopterygians (Polypteridae, holostean, teleosts). Black frame highlights orthologous TRPV genes between vertebrate species. Blue arrows indicate TRPV-specific local gene duplication and black cross, gene missing. TRPV5 has been duplicated repeatedly and independently in mammalian, sauropsid, amphibian, chondrichthyan, Polypteridae, Esocidae and cyclostome lineages. Mammalian-specific TRPV5 duplicated paralogs are classically referred to as TRPV5 and TRPV6. One of the sauropsid-specific TRPV5 paralog is undergoing pseudogenization in birds. Amphibian (anuran)-specific serial gene duplication of TRPV5 led to four paralogs. Two of the three duplicated chondrichthyan-specific paralogs are translocated. Serial duplication of TRPV5 in Polypteridae led to up to five paralogs. Duplicated TRPV5 paralogs specific of each lineage were located next to each other, reflecting local gene duplications, except in chondrichthyans where two of the three chondrichthyan-specific TRPV5 paralogs were translocated. The TRPV5 genomic region has been duplicated *via* the teleost-specific whole genome duplication (3R), but a single TRPV5 paralog is present in extant teleosts suggesting an early loss of one paralog after the 3R. The genomic region has been further duplicated *via* the salmonid-specific whole genome duplication (4R) leading to duplicated paralogs of TRPV5 (TRPV5α and TRPV5β). See **Supplementary Table S2** for TRPV and neighboring genes sequences accession numbers. Coelacanth scaffolds: JH127875/JH128489/JH127645/JH127253. Sea lamprey scaffolds: Chr 28/Chr 39/75 Unlocalized Scaffold.

We compared TRPV5/6 genomic regions in several vertebrate genomes: a cyclostome (sea lamprey), a chondrichthyan (spotted catshark), four sarcopterygians (human, duck, Western clawed frog and coelacanth), two non-teleost actinopterygians (reedfish and spotted gar), and five teleosts (European eel, medaka, zebrafish, northern pike and Atlantic salmon) (**Figure 7** and **Supplementary Table S2**). The duck genomic region was used as a template because in mammals including human this region has been largely rearranged as compared to the other vertebrates. Ten neighboring genes were identified and conserved in most of the mentioned genomes: AICDA, SLC2A3, FOXJ2, VAMP1, GNB3, FAM131B, CLCN1, CASP2, KEL, EPHB6, supporting orthology of TRPV5 among gnathostomes and possibly extending even to lamprey. Duplicated TRPV5 paralogs were found in tandem in human, duck, and northern pike. Despite the similar tandem position of the TRPV5 duplicates in the various vertebrate groups, the sequence-based phylogenetic tree (**Figure 6**) shows that these local gene duplications most likely occurred independently in each of these lineages. In the Western clawed frog, serial gene duplications led to four paralogs located side by side. In the reedfish, the five paralogs are also located adjacent to one another, supporting origin by serial gene duplications. In the catshark, where three chondrichthyan-specific TRPV5 paralogs are present, one paralog is located in a genomic region orthologous to TRPV5 of the other gnathostomes, while the others are on a separate chromosome, presumably as a result of translocation. Surprisingly, we found that it is the TRPV5 paralog at the conserved synteny position that has been lost in some species (elephant shark and whale shark, data not shown). In the sea lamprey, the current status of the genome with small scaffolds did not allow us to see if the two cyclostome-specific TRPV5 paralogs are on the same chromosome or not.

In teleosts, all species displayed duplicates of the following four TRPV5 neighboring genes: SLC2A3, GNB3, FAM131B and CLCN1 (**Figure 7**). In contrast, the reedfish and the spotted gar possessed only a single copy of these genes, supporting origin of the duplicated region in teleosts by 3R. Only a single TRPV5 gene has been identified in most teleosts investigated in this study, including representatives of basal lineages (European eel and Asian bonytongue). This TRPV5 gene is located in the same paralogon member as shown by the single conserved paralogs of the neighboring genes AICDA, FOXJ2, CASP2, KEL and EPHB6 in addition to the conserved duplicated paralogs of SLC2A3, GNB3, FAM131B and CLCN1. The other TRPV5 gene resulting from teleost 3R, the one that has been lost shortly after 3R, would have been located in the related synteny region. As mentioned above, the northern pike has a more recent local gene duplication of the single teleost TRPV5. In the Atlantic salmon, four copies exist of SLC2A3 and GNB3, and three copies of FAM131B and CLCN1 (**Figure 7**), as a result of the salmonid-specific 4R event. We propose the names TRPV5α and TRPV5β for the duplicates resulting from 4R. A previous study in another salmonid, the rainbow trout, identified only a single TRPV5 gene (also named ECaC) and showed expression in the gill (49). Our present finding of two TRPV5 genes in salmonids should stimulate investigation of their respective roles.

#### 3.4.3 Phylogeny of TRPV7 and TRPV8

Both of these subtypes are present in species representing cyclostomes, chondrichthyans and sarcopterygians (**Figure 6** and **Supplementary Table S1**). Some of the sequences that we have identified as either TRPV7 or TRPV8 have been previously annotated as TRPV5, TRPV6, TRPV5-like or TRPV6-like by NCBI and/or Ensembl databases but our phylogenetical and synteny studies showed that they were not orthologous to vertebrate TRPV5/6. The lamprey TRPV7 and TRPV8 sequences diverged at the base of each gnathostome TRPV7 and TRPV8 clades, respectively, in agreement with vertebrate phylogeny and highlighting the ancient origin in vertebrates of these two types.

Among chondrichthyans, the holocephalan possesses one TRPV7 and one TRPV8 gene, while elasmobranchs have one TRPV7 and two TRPV8 genes. The holocephalan TRPV8 sequence branched at the base of the elasmobranch TRPV8-1 and TRPV8-2 sister clades (**Figure 6**), suggesting an elasmobranch-specific duplication of TRPV8.

In sarcopterygians, TRPV7 and TRPV8 are present in the actinistian (coelacanth), previously annotated as TRPV6-like and TRPV5-like, respectively. In the amphibian *X. tropicalis*, TRPV7 and TRPV8 genes that we identified in the present study have been previously annotated as RBM19 (RNA Binding Motif Protein 19) and TULP2 (TUB Like Protein 2), respectively, in the Ensembl database, and as TRPV6 in the NCBI database. As for actinistian and chondrichthyan species, our phylogenetic and synteny analyses allowed us to identify these genes as orthologs of the TRPV7 and TRPV8 subtypes. 20 reported a gene in Western clawed frog that they called TRPV8, which corresponds to TRPV7 in our analyses.

In a prototherian mammal, the platypus, one non-annotated sequence manually reconstructed in the present study clustered with the TRPV7 subtype in the phylogenetic analysis. Another platypus sequence, non-annotaded in Ensembl database and annotated as TRPV6-like in the NCBI database, clustered with TRPV8 in our tree (**Figure 6**). 20 reported three novel TRPV genes in platypus that they named TRPV7, TRPV8 and TRPV9. These correspond to TRPV3, TRPV7 and TRPV8, respectively, in our phylogenetic analysis. No TRPV7 sequence was found in either Metatheria or Eutheria, suggesting loss of TRPV7 gene in the common ancestor of these lineages after the emergence of prototherian (**Supplementary Figure S2C**). In the opossum, BLAST analyses allowed us to retrieve one non-annotated short sequence (1 exon), which clustered with the TRPV8 subtype in the phylogenetic analysis (**Figure 6**), and could reflect pseudogenization of TRPV8 in metatherian mammals. BLAST search in eutherian genomes did not reveal any TRPV8 sequences, indicating complete loss of TRPV8 in the ancestor of eutherian mammals.

In sauropsids, neither TRPV7 nor TRPV8 genes have been found in any species studied, including squamate, chelonian, crocodilian and bird species, suggesting that both TRPV7 and TRPV8 have been lost in the ancestor of the sauropsid lineage.

In actinopterygians, one previously non-annotated gene was retrieved by BLAST searches in Polypteridae (reedfish) that clustered with the TRPV7 clade. Another reedfish sequence, annotated as TRPV6-like in NCBI database, clustered with the TRPV8 clade. BLAST analyses did not reveal any TRPV7 nor TRPV8 sequences in any other actinopterygians investigated such as holostean (spotted gar) and teleosts, suggesting loss of both TRPV7 and TRPV8 in the neopterygian common ancestor of holosteans and teleosts after the divergence of Polypteridae (**Supplementary Figure S2D**). All these conclusions are consistent with the results from the synteny analysis (see section 3.4.4.).

#### 3.4.4 Conserved synteny of the TRPV7, 8 genomic region

**Figure 8:**
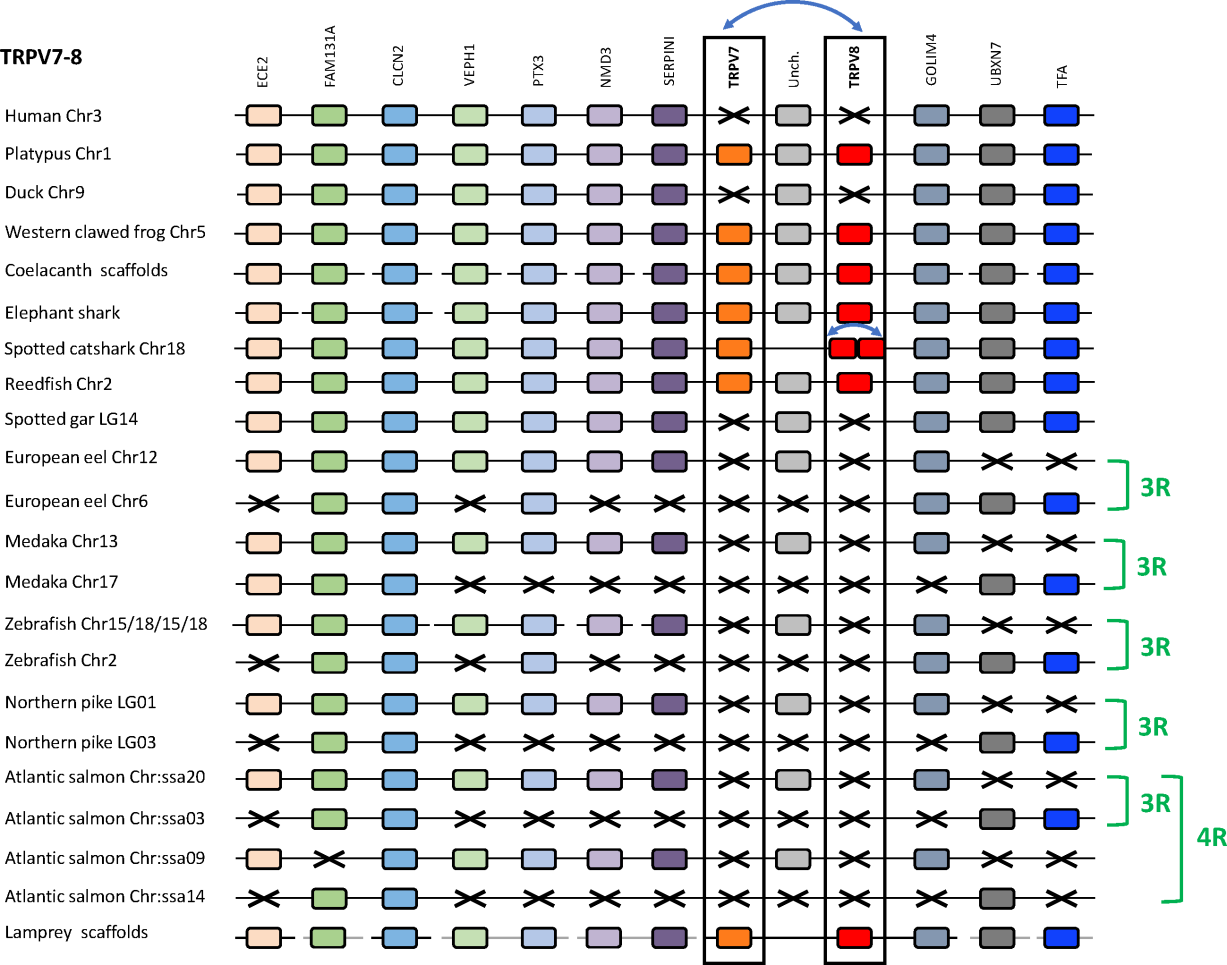
Conserved synteny between vertebrate TRPV7, 8 genomic regions. Platypus TRPV7, 8 genomic region is used as template. Eleven neighboring genes are shown. Gene colors are applied in order to show conserved synteny as well as sequence homology between representative vertebrate species: sarcopterygians (mammals, sauropsid, amphibian, actinistian), chondrichthyans, actinopterygians (Polypteridae, holostean, teleosts), cyclostome. Black frames highlight orthologous TRPV genes between vertebrate species. Blue arrows indicate TRPV-specific local gene duplication and black cross, gene missing. TRPV7 and TRPV8 genes are located on a close genomic region, reflecting an ancient local gene duplication and are conserved from cyclostomes to chondrichthyans and basal actinopterygians (Polypteridae) as well as to sarcopterygians up to prototherian mammals. TRPV7 and TRPV8 genes have been lost repeatedly and independently in various lineages: neopterygian actinopterygian, sauropsid and therian mammalian lineages. The TRPV7, 8 genomic region has been duplicated *via* the teleost-specific (3R) and salmonid-specific (4R) whole genome duplications. As the loss of TRPV7 and TRPV8 genes in a neopterygian ancestor predates the emergence of teleosts, there was no impact of 3R and 4R on these genes. See **Supplementary Table S2** for TRPV and neighboring genes sequences accession numbers. Coelacanth scaffolds: JH126892/JH129010/JH126572/JH127331/JH127319/JH127370/JH128935/JH128336. Elephant shark scaffolds: KI635855/KI635949/KI636688.1. Sea lamprey scaffolds: Chr55/Chr 21/Chr 67/Chr 6/Chr 28/Chr 5/Chr 39.

We compared TRPV7, 8 genomic regions in one cyclostome (sea lamprey), two chondrichthyans (elephant shark and spotted catshark), five sarcopterygians (human, platypus, duck, Western clawed frog and coelacanth), two non-teleost actinopterygians (reedfish and spotted gar), and five teleosts (European eel, medaka, zebrafish, northern pike and Atlantic salmon) (**Figure 8** and **Supplementary Table S2**). Platypus was used as template as TRPV7 and TRPV8 are missing in human. When present, TRPV7 and TRPV8 were located in the same genomic region, either as a tandem pair in cyclostomes (sea lamprey) and chondrichthyans (elephant shark and spotted catshark), or separated only by a single gene “uncharacterized” supporting origin by a local gene duplication. The two copies of TRPV8 in the spotted catshark were located next to each other (**Figure 8**), indicating an additional duplication of TRPV8 in elasmobranchs.

Eleven neighboring genes to TRPV7 and TRPV8 with conserved synteny could be identified in the genomes of the vertebrate species investigated in this study: ECE2, FAM131A, CLCN2, VEPH1, PTX3, NMD3, SERPINI, GOLIM4, UBXN7, TFA and one gene called “uncharacterized” in NCBI. We were able to retrieve previously non-identified TRPV neighboring genes in genome databases using the TBLASTN algorithm of the Ensembl Genome Browser website.

The “uncharacterized” gene is located between TRPV7 and TRPV8 in platypus, a position observed in the other sarcopterygians (coelacanth, amphibians), as well as in the actinopterygian Polypteridae (reedfish) (**Figure 8**). SERPINI and GOLIM4 are next to TRPV7 and TRPV8, respectively, positions also conserved throughout vertebrates. In species lacking TRPV7 and TRPV8, SERPINI and GOLIM4 flank the “uncharacterized” gene. Opossum lacks TRPV7 but the TRPV8 pseudogene is still present and located in a conserved position between the “uncharacterized” gene and GOLIM4 (not shown). In chondrichthyans (elephant shark), the “uncharacterized” gene is not located between TRPV7 and TRPV8, but on the opposite site of TRPV7. We hypothesize that this reflects the ancestral organization, and that the change of position of the “uncharacterized” gene may have led to disruption of the tandem TRPV7/TRPV8 pair in the ancestor of osteichthyans. Analyses of the “uncharacterized” gene in genomes of species investigated allowed us to determine that it is a leucin-rich gene. The conservation of this “uncharacterized” as well as other TRPV close neighboring genes such as fam, shpk, flot2, p2rx5, ephb6, m6pr, casp2, clcn, ece2, kel, gltp, git2, ssh, myoc, emc6, serpini or golim4 throughout vertebrate evolution suggests a strong evolutionary pressure and raises the question of their potential functional roles in relation to TRPVs.

The conservation and close proximity of SERPINI, the “uncharacterized” gene and GOLIM4, even in species lacking TRPV7 and TRPV8, such as holosteans, teleosts, sauropsids and eutherian mammals, supports repeated losses of TRPV7 and TRPV8 in these lineages.

Two of the TRPV7, 8 neighboring genes, FAM131A and CLCN2, are duplicated in all of the investigated teleosts and are syntenic with one another (**Figure 8**). Two more syntenic duplicates are present in some of the species, PTX3 and GOLIM4. The non-teleost actinopterygians (reedfish and spotted gar) possess only a single copy of these genes, supporting duplication of this region in teleost 3R. Atlantic salmon has four copies of CLCN2 and three copies of FAM131A, and these duplicates are syntenic with each other, consistent with origin by salmonid 4R. As TRPV7 and TRPV8 were lost before the teleost radiation, teleost 3R and salmonid 4R had no effect on their copy number.

#### 3.4.5 Paralogon for vertebrate TRPV5/6, 7, 8 genes

**Figure 9:**
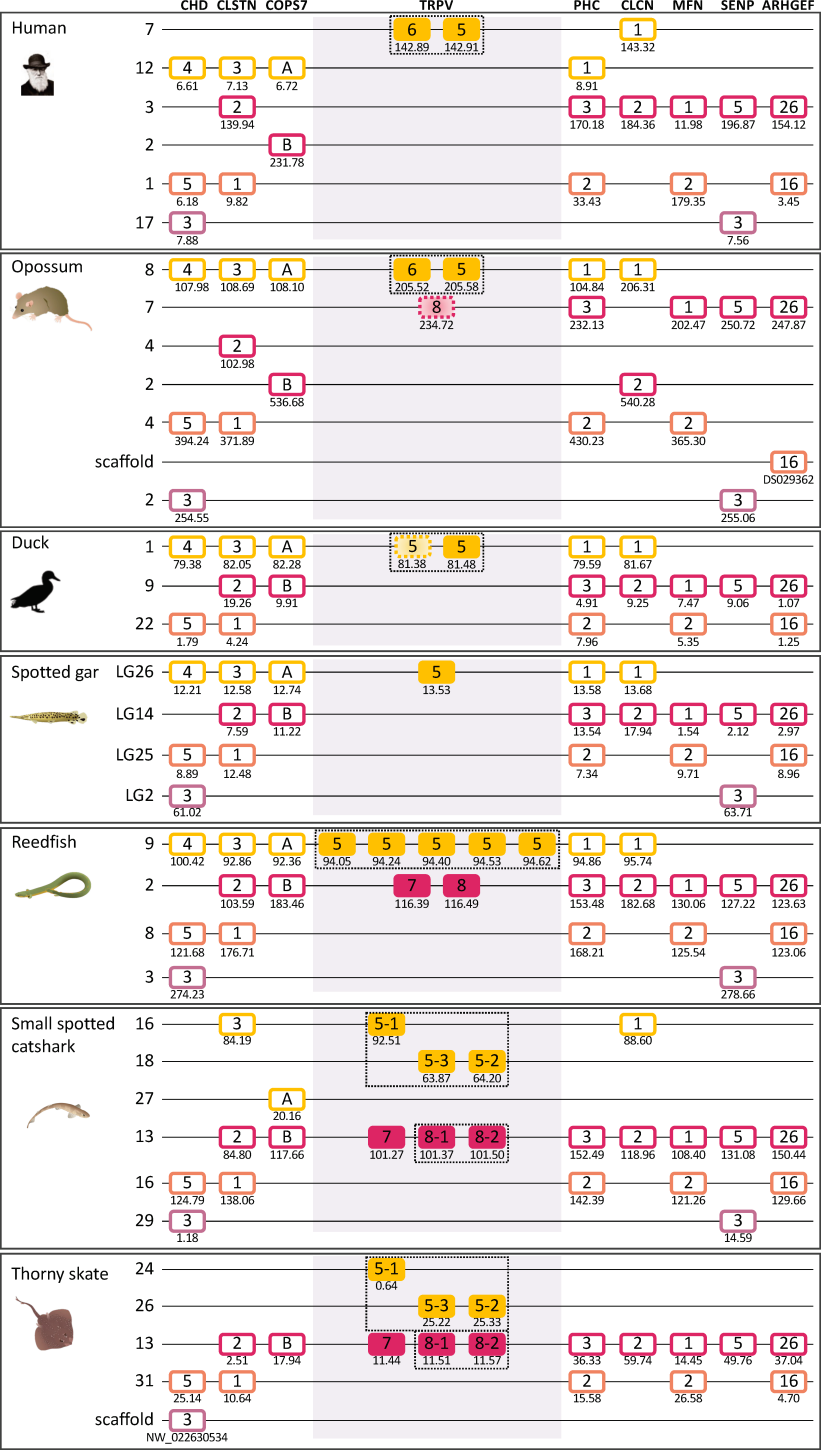
Paralogon for vertebrate TRPV5,6,7,8 genes. Gnathostome chromosomal regions harboring the genes of the TRPVD subfamily: the TRPV genes 5 and 6 are in a chromosomal region with several neighboring genes that have related genes also on the chromosome where TRPV genes 7 and 8 are located, as well as two additional chromosomes which lack TRPV genes. Sequence-based phylogenetic analyses (**Supplementary Figure S4**) show that all of these gene families underwent duplications in time period of the WGD events 1R and 2R. Thus, these chromosomal regions are in agreement with quadruplication of an ancestral gnathostome (or vertebrate) chromosome. The species shown are human, duck (*Anas platyrhynchos*), spotted gar (*Lepisosteus oculatus*), reedfish (*Erpetoichthys calabaricus*), small spotted catshark (*Scyliorhinus canicula*), and thorny skate (*Amblyraja radiata*). The number below each gene shows the position in the chromosome. The order of the genes along the chromosomes has been re-shuffled to highlight the similarities. Animal illustrations are used with permission from Daniel Ocampo Daza, source: www.egosumdaniel.se, except the human image which is used with permission from https://commons.wikimedia.org and the duck image which is from http://phylopic.org.Figure.

Although TRPV5/6 and TRPV7, 8 are present on two separate chromosomes in all species investigated, their respective chromosomal regions were found to share representatives from two other gene families, namely CLCN (voltage-dependent chloride channel) and FAM131, see **Figures 7**, **8**. This discovery invited a search for additional shared neighboring gene families. As shown in **Figure 9** and further described in section 3.5, we could identify in total eight neighboring gene families with either three or two members, together defining a quartet of chromosomal regions, thus a paralogon consistent with chromosome quadruplication in 2R.

The overall picture thereby suggests that TRPV5 and TRPV7 arose from a common ancestral TRPV gene, TRPVD, by chromosomal duplication, most likely in the 1R WGD event. Then TRPV7 generated a local duplicate before the gnathostome radiation that became TRPV8. At least one TRPV5 ortholog is present in every gnathostome species investigated, and furthermore the TRPV5 gene was duplicated locally on multiple independent occasions in various lineages, and the duplicate in mammals has been previously named TRPV6.

### 3.5 Analysis of synteny blocks and paralogons

As described above, the sequence-based phylogenetic analyses (**Figure 1**) clearly divide the gnathostome TRPV genes into two subfamilies, one with the descendants of the clade C ancestor, i.e. TRPV1, 2, 3, 9 and TRPV4, located on two separate chromosomes (**Figures 3**–**5**), and the other subfamily originating from the clade D ancestor, namely TRPV5/6 and TRPV7, 8, on two additional chromosomes (**Figures 7**–**9**). The extended synteny analyses show that the two chromosomal regions for the members of the TRPVC subfamily share several neighboring gene families (Section 3.3.5 and **Figure 5**). Thus, these two chromosomal regions are related to one another. Likewise, the two chromosomal regions for the members of the TRPD subfamily were found to resemble each other (Section 3.4.5 and **Figure 9**), but had no similarity to the chromosomal regions for the clade C subfamily. Below we describe in detail how the chromosomal regions for each subfamily were analyzed and the results leading to the conclusion about chromosome duplications.

Using the spotted gar chromosomes as starting point, we examined neighboring genes within 10 Mb on either side of the TRPV genes (**Figures 5**, **9**). We then searched for additional members of these neighboring families, aligned the sequences and performed phylogenetic analyses (see **Supplementary Figures S3** and **S4**). The gene families with appropriate outgroup sequences from more distantly related species all displayed phylogenies and species distribution that showed duplications in the same time period as the basal gnathostome WGD events (2R).

Furthermore, the neighboring gene families extended each set of related chromosomal regions from two to four members, emphasizing the four-fold symmetry resulting from 2R, in other words a paralogon (i.e., a set of related chromosomal regions) (**Figures 5**, **9**). In some species, chromosomal rearrangements have obscured the similarities between the members of a paralogon, but by taking into consideration all of the species representing the major gnathostome lineages, the four-fold pattern clearly emerges for the chromosomal regions of each of the TRPV clades, although the TRPV genes themselves are present on only two of the four chromosomes in each paralogon. The species that are particularly useful for deducing the ancestral chromosomal organization are those that seem to have undergone few rearrangements and thus have a very stable chromosomes, especially reedfish and spotted gar. The teleost 3R and the salmonid 4R events have been previously shown to result in paralogons with up to 8 or 16 members, respectively (36, 45). We included teleosts in synteny analyses of each TRPV (**Figures 3**, **4**, **7**, **8**) which highlighted the impact of 3R and 4R, but we did not include teleosts in the present paralogon analyses.

The paralogon that contains the TRPVC subfamily members, TRPV1, 2, 3, 9 and TRPV4, has three full quartets as neighbors (DOC/RPH3A, ATP2A and CORO) and eight families that are triplets (**Figure 5**) when taking into consideration all species that were investigated in detail with regard to gene neighbors. Each of the neighboring gene families was analyzed for sequence-based phylogeny (**Supplementary Figures S3**) and was found to display gene duplications in the same time frame as the two basal gnathostome WGD events. Together these gene families with quartets and triplets comprise four chromosomal regions that are consistent with quadruplication in 2R (**Figure 5**). Some chromosomal rearrangements have taken place, translocating a few genes for instance in human, and some genes have been lost in all analyzed species, most likely in the common ancestor of the gnathostomes, but some may have been lost independently in different lineages. Nevertheless, the overall picture that emerges is one of fourfold chromosomal symmetry. The gene repertoire and configuration is especially well conserved in the reedfish, whereas the bird representative, the duck, has lost almost all of one of the four chromosomal regions and most of another one. The most complete gene repertoires for this part of the paralogon are found in human (and other mammals) and reedfish, both of which have 35 genes in the 11 neighboring families. The smallest number is found the thorny skate with only 23, small spotted catshark with 24 and duck with 26. It remains possible that a few more genes exist in some of the species but have not yet been covered by the genome assemblies.

A separate paralogon was identified for the two chromosomal regions with TRPVD subfamily members TRPV5/6 and TRPV7, 8 (**Figure 9**). These chromosomal regions share eight neighboring gene families, three of which have three members in most of the species investigated (CLSTH, CHD3/4/5 and PHC) and five families consist of two members (CLCN, COPS7, MFN, SENP3/5 and ARHGEF16/26). The chromosomal locations of these neighboring genes together define a quartet of chromosomal regions, thus a paralogon, and the phylogenetic trees of the gene families are consistent with duplications in the 2R time range, as further supported by the species distribution (**Supplementary Figure S4**). This paralogon is less complete than the one for the clade C. TRPV genes (**Figure 5**) and some chromosomal rearrangements have taken place here too, but the overall organization is consistent with a total of four related chromosomal regions. Again, the reedfish has the most complete repertoire with a total of 19 of the original genes still present, followed by 18 in several other lineages. The lowest number is displayed by the thorny skate with 13. Note that FAM131 (**Figures 7**, **8**), which is a triplet in some of the species including human, was not included in the paralogon analysis in **Figure 9** due to lack of a suitable outgroup to root the tree, hence making it difficult to determine the time point for its triplication.

## 4 Evolution of the TRPV family

**Figure 10:**
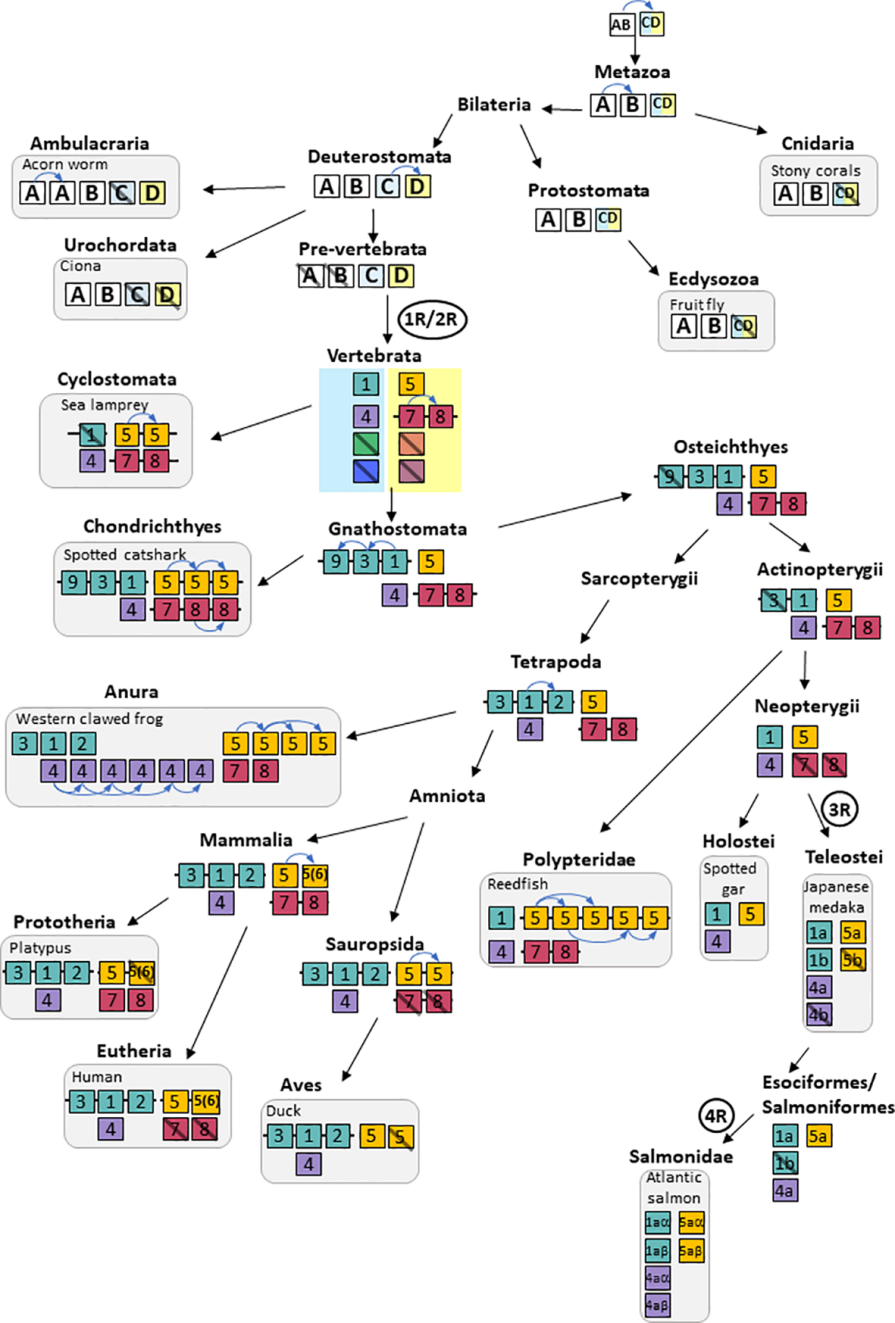
Proposed evolutionary scenario for the TRPV family. Local TRPV gene duplications led to three genes in early metazoans that we have named A, B, and C/D. The C/D gene was lost independently in the cnidarian and ecdysozoan lineages. An additional duplication of the C/D gene led to a total of four TRPV genes in early deuterostomes. The TRPVC gene was lost in ambulacrarians and TRPVC and TRPVD were lost in urochordates, while the TRPVA and B genes were lost in pre-vertebrates after the divergence of urochordates. The TRPVC and D genes were duplicated in the 1R/2R whole-genome duplications (WGD) and together with local duplications gave rise to the TRPV types 1, 2, 3, 4, 9 and 5/6, 7, 8 respectively, present in jawed vertebrate lineages. In the vertebrate ancestor, the empty boxes with a diagonal bar represent the additional genes that were probably generated in 1R/2R but that were lost early in the vertebrate lineage. The colors used for vertebrate TRPV are consistent with the colors used in paralogon block figures 5 and 9. Deduced TRPV gene duplications are shown in the main metazoan lineages in relation to the main WGD events, i.e., the vertebrate 1R and 2R, the teleost 3R and the salmonid 4R, as well as the multiple independent lineage-specific TRPV gene duplications (blue arrow) or losses (black bar). The lineages are represented by key species. The scheme is based on protein sequence phylogeny, species distribution, conserved synteny and paralogon analyses, where the two latter types of information also include numerous gene families located adjacently on these chromosomes.

Our discoveries of distinct previously undescribed TRPV family members in a variety of metazoans and especially gnathostomes has allowed us to propose a comprehensive evolutionary scheme outlined in detail in **Supplementary Figure S2** and summarized in **Figure 10**. Our global phylogenetic analysis includes representatives of key groups of metazoans and suggests that three types of TRPV, that we have named TRPVA, TRPVB and TRPVC/D, would have been already present in an ancestral metazoan (**Supplementary Figure S2A** and **Figure 10**). TRPVA and TRPVB have been retained in cnidarians, protostomes and non-vertebrate deuterostomes, but have been lost in vertebrates. Lineage-specific events generated paralogs of TRPVA independently in hemichordates, echinoderms and mollusks and of TRPVB in nematodes. TRPVC/D seems to have been lost independently in cnidarians and ecdysozoans, whereas it has been lineage-specifically duplicated in lophotrochozoan annelids and mollusks. A duplication of TRPVC/D gave rise to TRPVC and TRPVD, which probably happened in an ancestral deuterostome. TRPVC was independently lost in ambulacraria, cephalochordates and urochordates, while TRPVD was independently lost in echinoderms and in urochordates (**Supplementary Figure S2A** and **Figure 10**). TRPVC and TRPVD genes are at the origin of all the vertebrate TRPV types.

In vertebrates, our synteny and paralogon analyses allow us to propose that 1R/2R WGD duplicated both TRPVC and TRPVD (**Figure 10**). TRPVC generated TRPV1 and TRPV4 while TRPVD gave rise to TRPV5 and TRPV7 whereupon the last-mentioned soon generated TRPV8 by local duplication. Local duplications of TRPV1 gave rise to TRPV3, TRPV9 in gnathostomes. Subsequently, numerous independent gains and losses occurred as shown in **Supplementary Figure S2** and **Figure 10**. Local duplication of TRPV1 in tetrapods gave rise to TRPV2. TRPV3 has been lost in actinopterygians. Lineage-specific duplications of TRPV5 occurred independently in various lineages such as cyclostomes, chondrichthyans, actinopterygian polypterids, amphibians, sauropsids and mammals (where the duplicate is known as “TRPV6”). The TRPV7 and 8 types identified in the present study have been retained in cyclostomes, chondrichthyans, actinistians, amphibians, prototherian mammals and actinopterygian polypterids, but lost independently in therian mammals, sauropsids and actinopterygian neopterygians. The TRPV9 gene identified in the present study has been retained only in chondrichthyans.

The teleost-specific 3R event duplicated TRPV1, 4 and 5 inherited from their neopterygian ancestor, but one duplicate was lost shortly after 3R for TRPV4 and TRPV5. Both 3R-duplicates of TRPV1 (TRPV1a and 1b) were conserved in various teleost species but TRPV1b was lost independently in some teleosts. Salmonid specific 4R WGD further duplicated TRPV1a as well as TRPV4 and TRPV5.

The highest number of TRPV family members among the vertebrate species investigated was found in the frog *Xenopus tropicalis* with as many as 15 TRPV genes and the lowest number of any vertebrate was found in the spotted gar with only three: TRPV1, 4 and 5. These three members were found in each and every gnathostome species investigated, implying that each carries out major and likely conserved functions. The many TRPV duplicates found in some lineages invites studies of the possible distinct roles of each family member.

In conclusion, our combined analyses of sequence-based phylogenies, species distribution, and in vertebrates also synteny and paralogons, has revealed a larger number of TRPV family members than previously reported, including the evidence of the three additional gnathostome subtypes TRPV7, TRPV8 and TRPV9 all of which are of early vertebrate origin. The TRPV family displays a highly dynamic evolution with a large number of lineage-specific events involving local gene duplications and gene losses. In the context of climate change and global warming, there is a growing interest for investigating TRPV in a variety of metazoan species of relevance for biomedical, agronomic or biodiversity conservation studies. The present work contributes to clarify the nomenclature of TRPV genes across metazoans and vertebrates. It provides necessary tools for predicting the number of TRPV types and paralogs according to vertebrate lineages, and for proposing a phylogenetically-based classification of TRPV across vertebrate species, compulsory to relevant inter-specific comparisons. This study opens new research avenues for the comprehensive investigation of the TRPV superfamily.

Information on the evolution of the repertoire of the TRPV genes in metazoans, distinguishing orthologs and paralogs, provides a useful classification for the scientific community, paving the way for detailed investigation of thermoreceptors in a diversity of biological models. So far only a few studies have analyzed the response to temperature of TRPV receptors outside eutherian mammals. Furthermore, our study reveals the presence of several additional TRPV paralogs, some of which are of ancient origin and some of which have subsequently been lost in some evolutionary lineages but have been retained in others. Knowledge about the complete TRPV repertoire is a prerequisite for future studies aiming at investigating their potential roles in thermosensing. Besides TRPV, other families of TRP channels (TRPM, TRPA and TRPC) have been reported to be involved in thermosensing (50). The present study describing the evolution of the TRPV family will be followed by studies of the other TRP families.

## Data availability statement

The original contributions presented in the study are included in the article/**Supplementary Material**. Further inquiries can be directed to the corresponding authors.

## Author contributions

MM, CB, JA, DL, and SD: designed the study. MM and CB: performed phylogenetic and syntenic analyses; drew the Figures. MM, CB, DL, and SD: analyzed the data. MM, DL, and SD: wrote the manuscript. MM, CB, JA, DL, and SD: reviewed the manuscript; approved the submitted version. All authors contributed to the article and approved the submitted version.

## Funding

MM is a recipient of France-Spain bilateral postdoctoral fellowship APOSTD (APOSTD/2020/053) from the Generalitat Valenciana and the European community. JA has funding from Spanish MICIU (Project EELGONIA; RTI2018-096413-B-I00). DL has funding from the Swedish Research Council and the Swedish Brain Foundation. SD has funding from MNHN and CNRS.

## Acknowledgments

We are grateful to Daniel Ocampo Daza for animal artwork, source: https://www.egosumdaniel.se.

## Conflict of interest

The authors declare that the research was conducted in the absence of any commercial or financial relationships that could be construed as a potential conflict of interest.

## Publisher’s note

All claims expressed in this article are solely those of the authors and do not necessarily represent those of their affiliated organizations, or those of the publisher, the editors and the reviewers. Any product that may be evaluated in this article, or claim that may be made by its manufacturer, is not guaranteed or endorsed by the publisher.

## Supplementary material

Supplementary Table 1 Accession numbers of TRPV sequences used for phylogeny analyses, with indication of current gene names in databases and of our proposed phylogenetically-based names (**Figures 1**, **2**, **6**).

Supplementary Table 2 Accession numbers and locations of TRPV and neighboring genes used for each TRPV synteny analyses (**Figures 3**, **4**, **7**, **8**).

Supplementary Table 3 Accession numbers and locations of TRPV and neighboring genes used for block synteny analyses (**Figures 5**, **8**)

Supplementary Figure 1 Global phylogeny of metazoan TRPV same as **Figure 1** but displaying species names and current gene names in databases.

Supplementary Figure 2 Detailed evolutionary scenario of TRPV family in metazoans. **(A)** early metazoans, cnidarians, bilaterians, protostomes and deuterostomes up to vertebrate ancestor. **(B)** cyclostomes, gnathostomes and chondrichthyans. **(C)** osteichthyans, sarcopterygians. **(D)** osteichthyans, actinopterygians. Blue arrow: local gene duplication. Red bar: gene loss. 1R/2R: vertebrate first round and second round of whole genome duplication (WGD). 3R: teleost third round of WGD. 4R: salmonid fourth round of WGD. Green arrow: duplication from WGD.

Supplementary Figure 3 Phylogeny of neighboring genes of TRPV1, 2, 3, 4, 9 block synteny analysis (**Figure 5**).

Supplementary Figure 4 Phylogeny of neighboring genes of TRPV5/6, 7, 8 block synteny analysis (**Figure 9**).
