# Supplementary figures and images for: Dynamic evolution of transient receptor potential vanilloid (TRPV) ion channel family with numerous gene duplications and losses
Source: Front Endocrinol (Lausanne). 2022 Nov 1;13:1013868. doi: 10.3389/fendo.2022.1013868 (PMC9664204; doi:10.3389/fendo.2022.1013868)

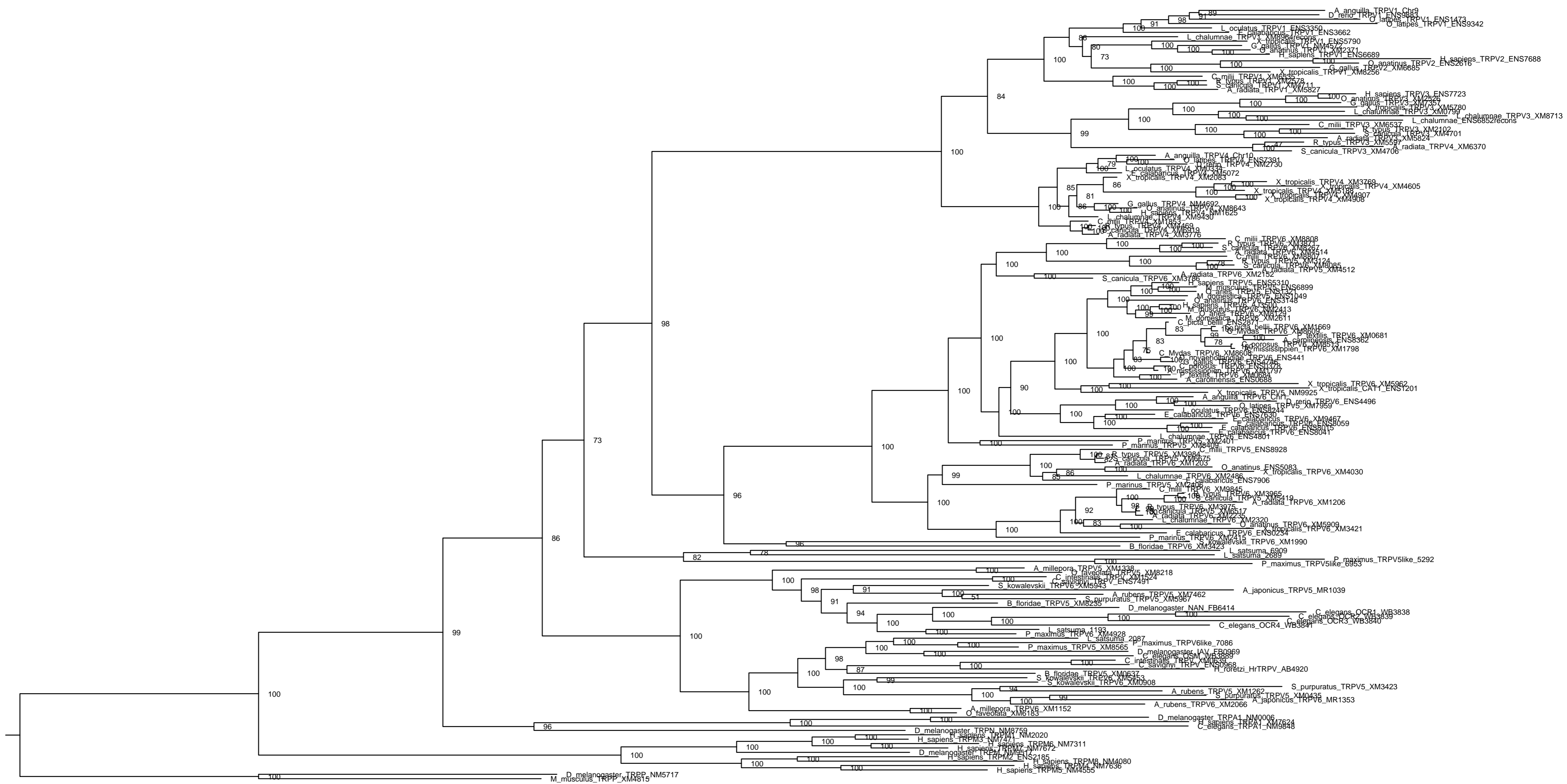

0.6

Supplement: Supplementary Figure 1 — Global phylogeny of metazoan TRPV same as Figure 1 but displaying species names and current gene names in databases. [file DataSheet_1.pdf]

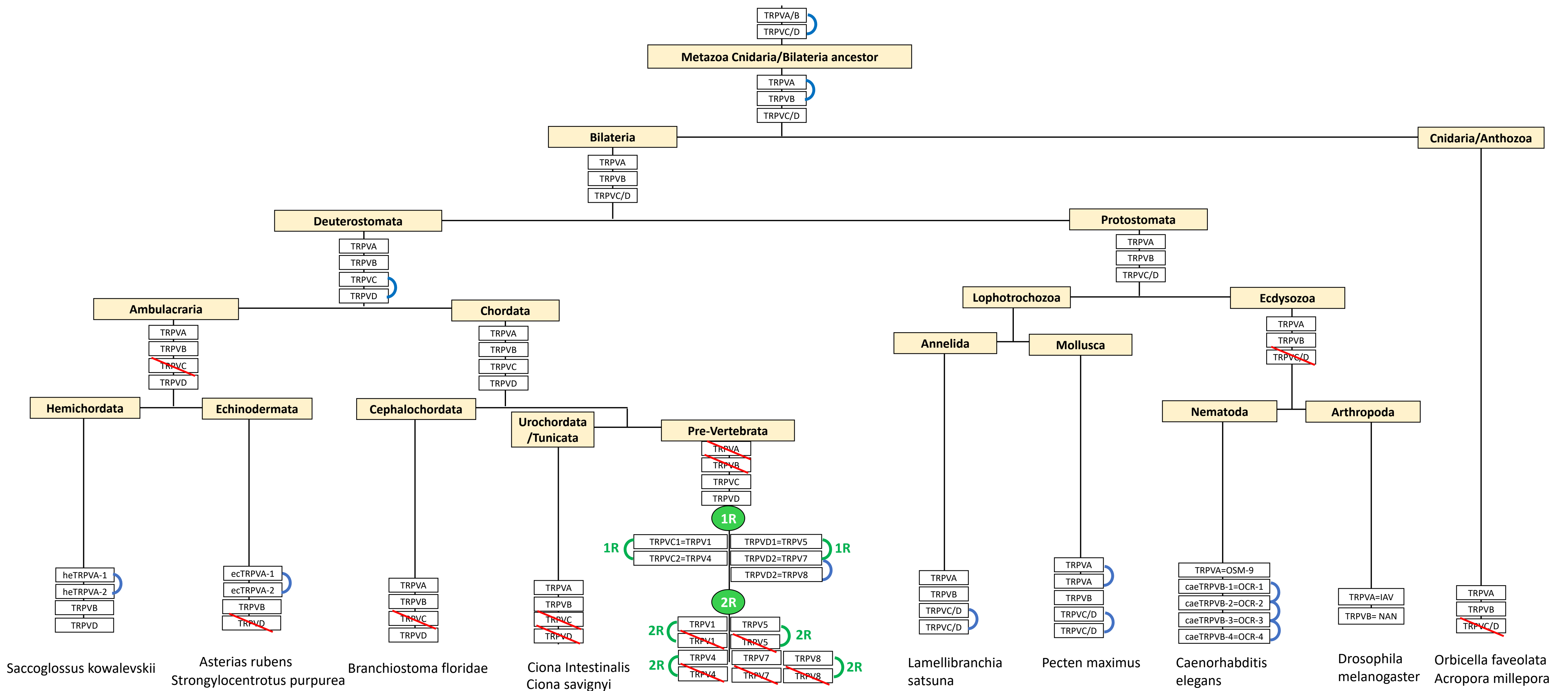

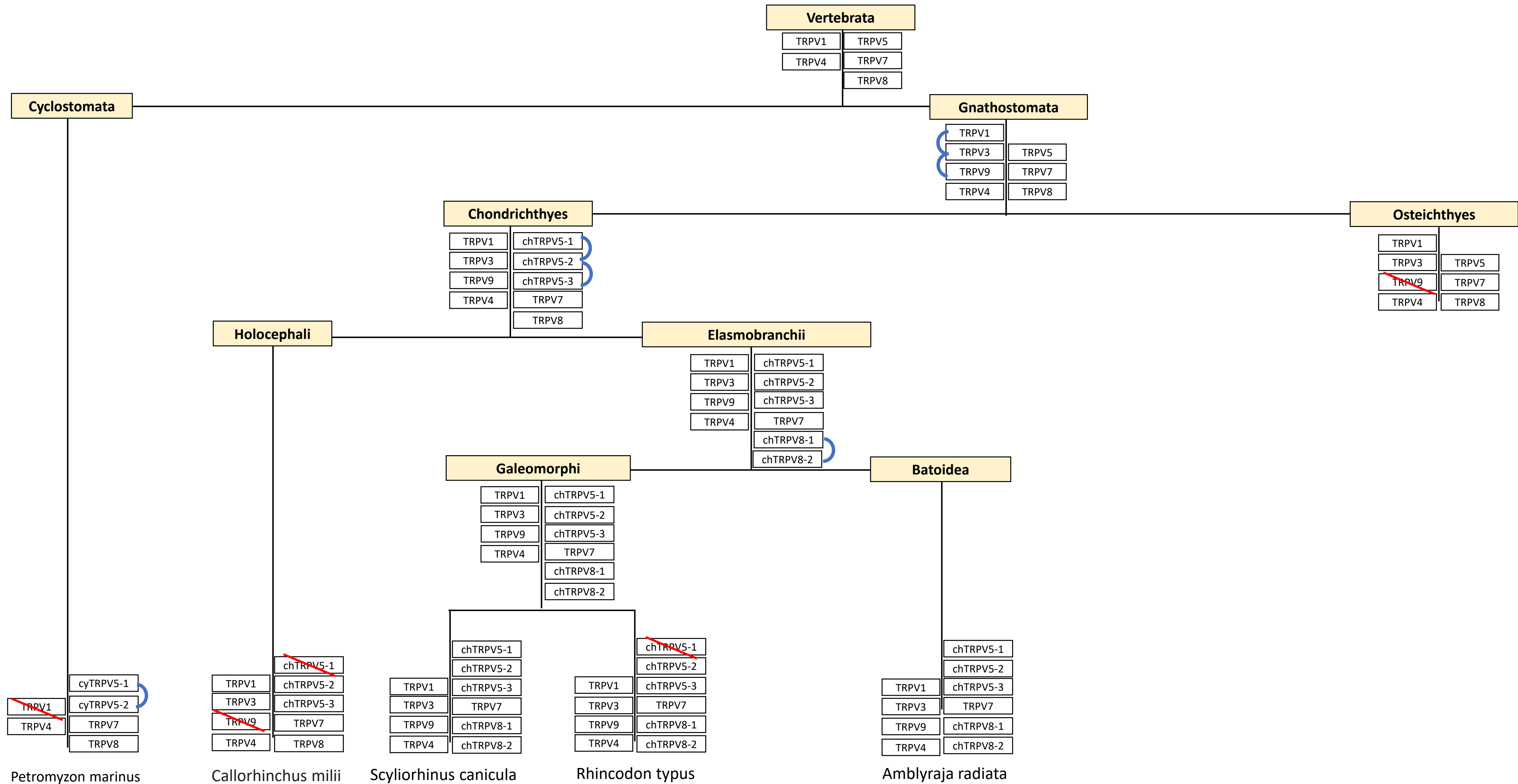

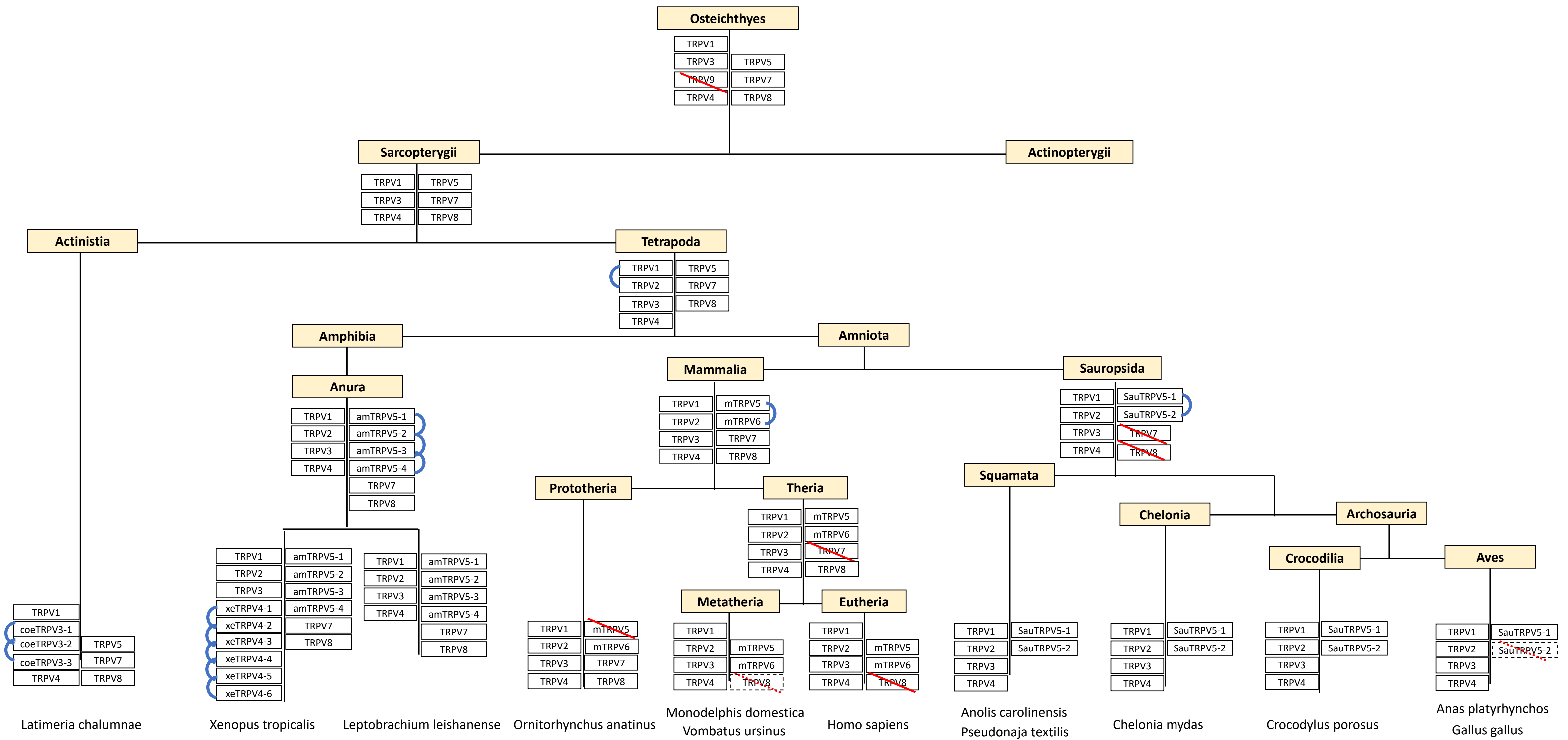

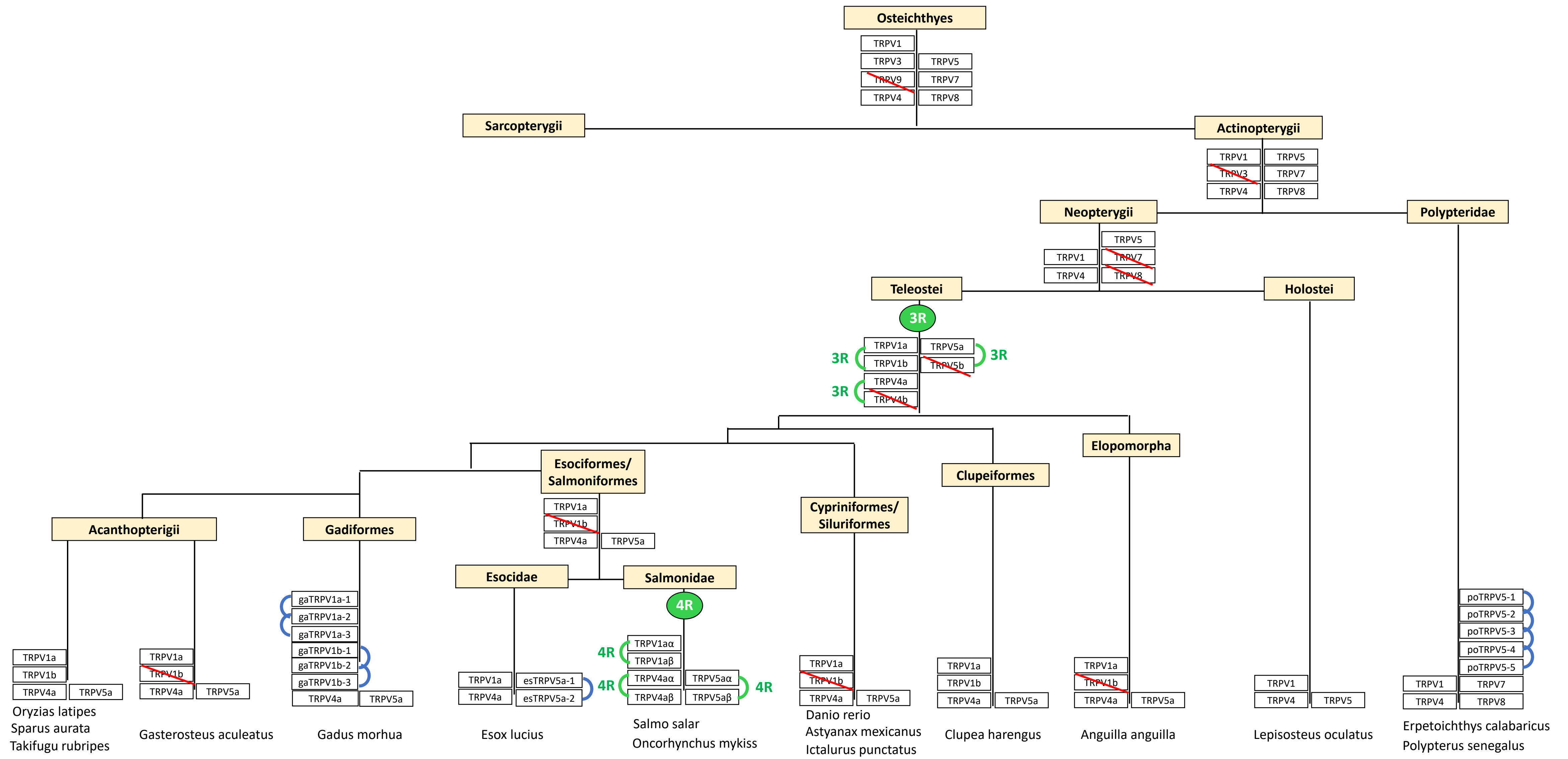

Supplement: Supplementary Figure 2 — Detailed evolutionary scenario of TRPV family in metazoans. (A) early metazoans, cnidarians, bilaterians, protostomes and deuterostomes up to vertebrate ancestor. (B) cyclostomes, gnathostomes and chondrichthyans. (C) osteichthyans, sarcopterygians. (D) osteichthyans, actinopterygians. Blue arrow: local gene duplication. Red bar: gene loss. 1R/2R: vertebrate first round and second round of whole genome duplication (WGD). 3R: teleost third round of WGD. 4R: salmonid fourth round of WGD. Green arrow: duplication from WGD. [file DataSheet_2.pdf]

Fig S3

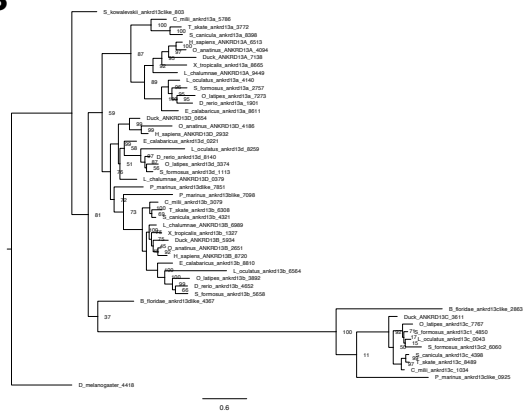

ANKRD

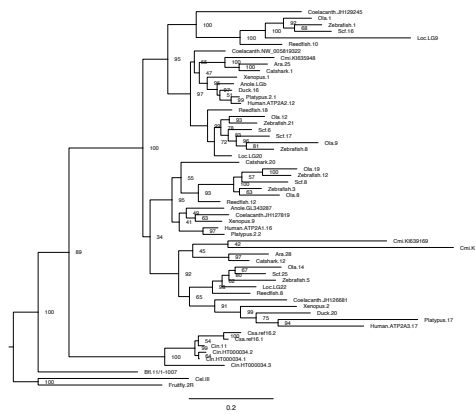

ATP2A

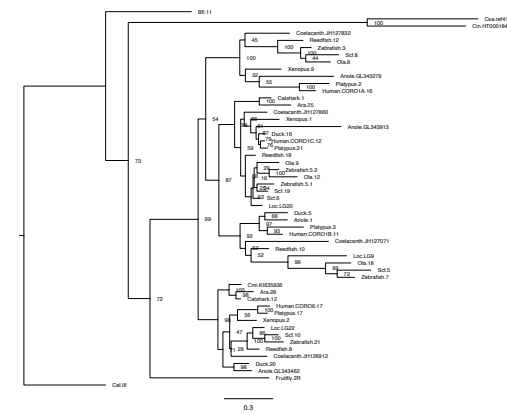

CORO

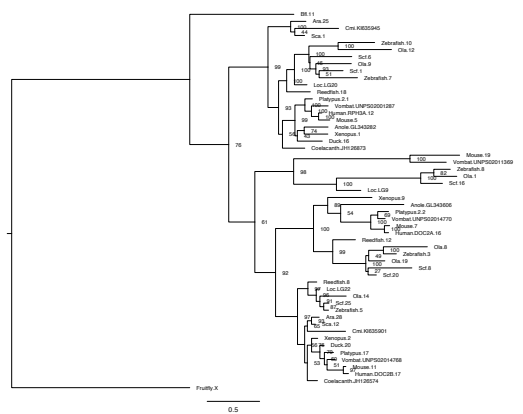

DOC2

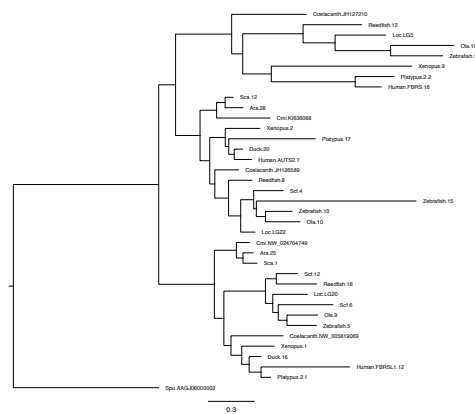

FBRS

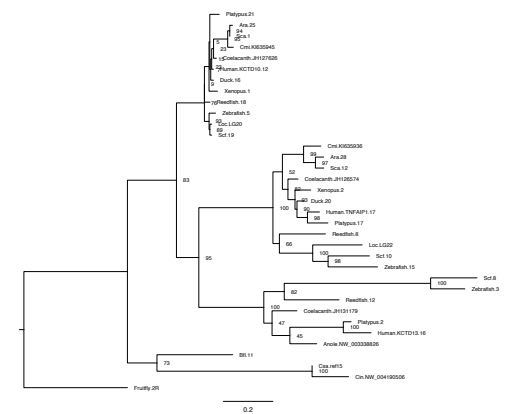

KCTD

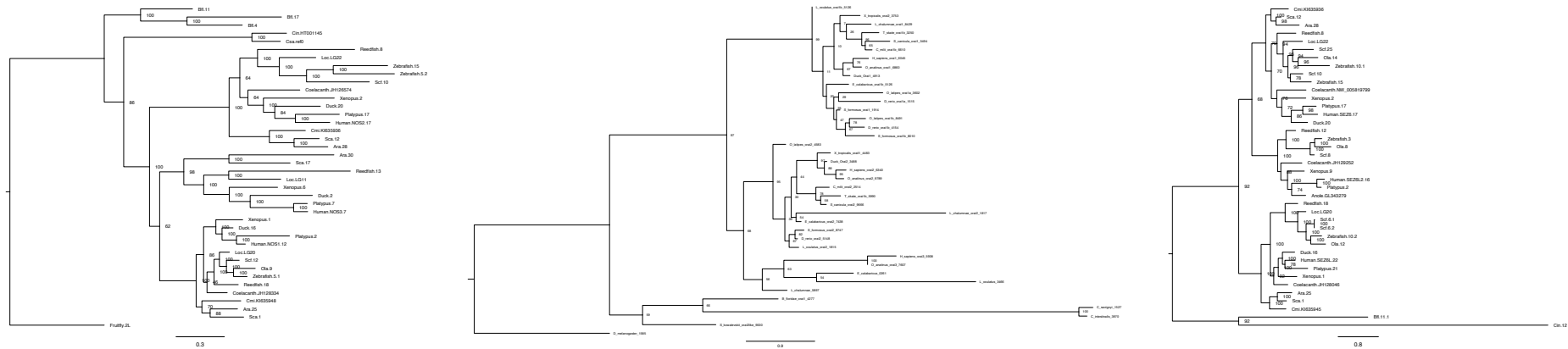

NOS

ORAI

SEZ6

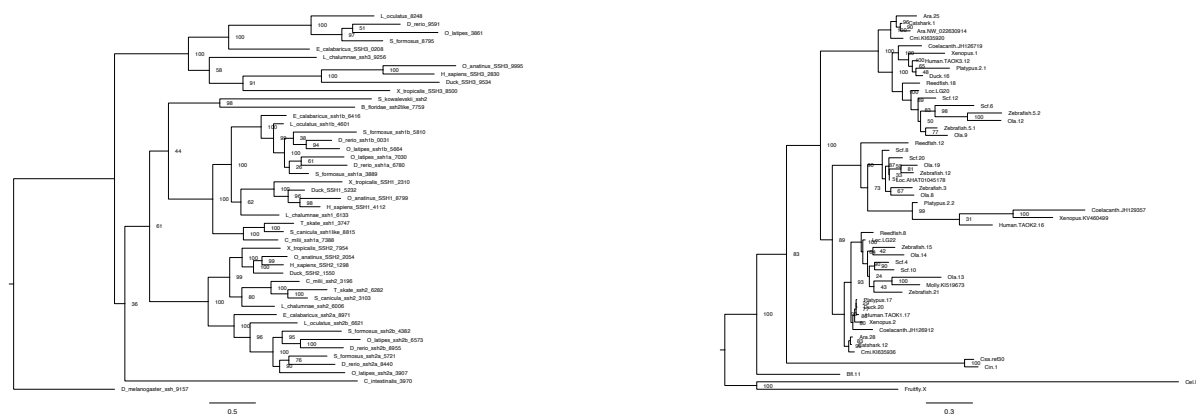

SSH

TAOK\_3

Supplement: Supplementary Figure 3 — Phylogeny of neighboring genes of TRPV1, 2, 3, 4, 9 block synteny analysis ( Figure 5 ). [file DataSheet_3.pdf]

**Fig S4**

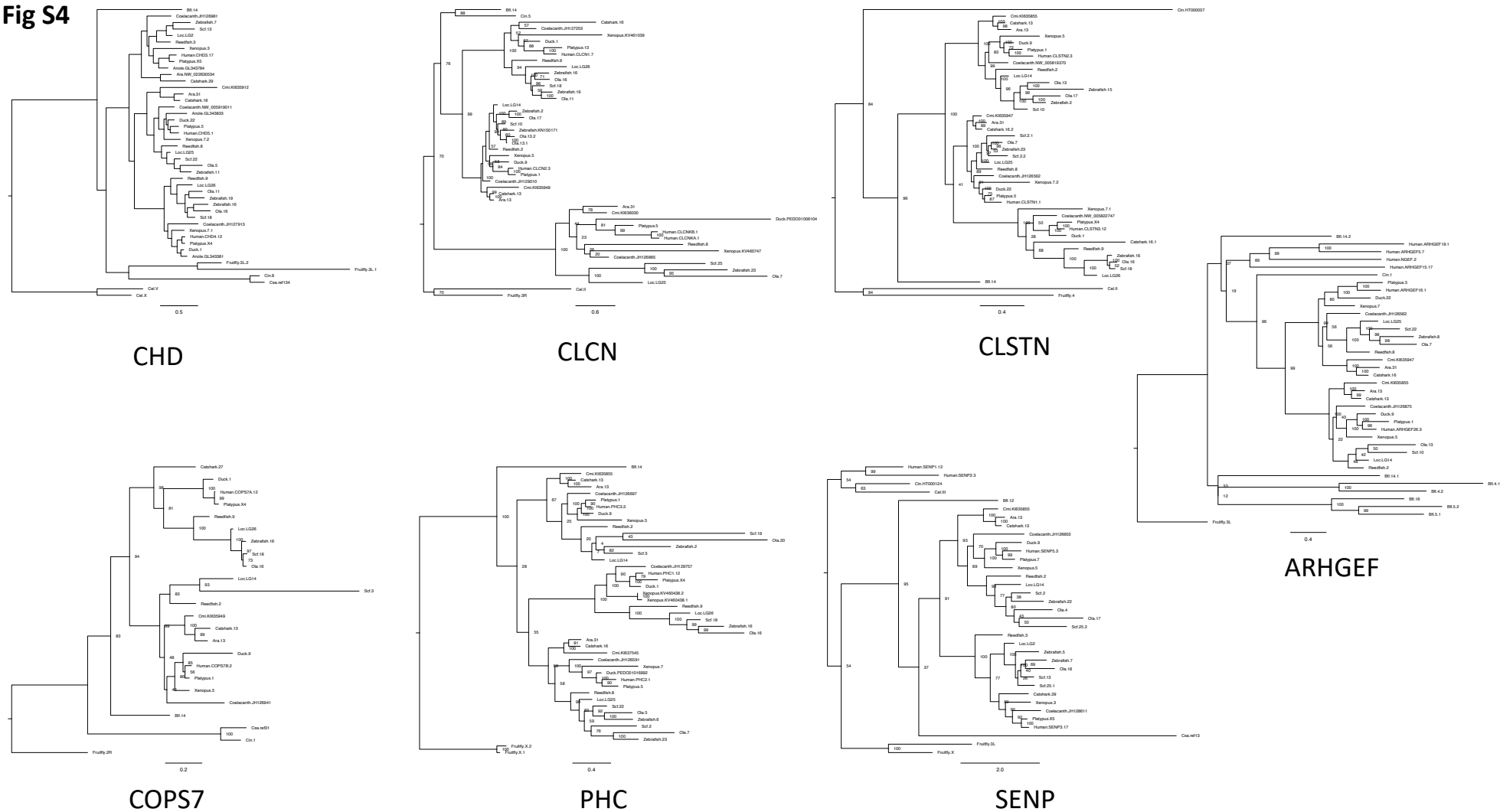

Supplement: Supplementary Figure 4 — Phylogeny of neighboring genes of TRPV5/6, 7, 8 block synteny analysis ( Figure 9 ). [file DataSheet_4.pdf]
